# Passive scalar transport in a cross-ventilating flow with upstream source: wind and water tunnel measurements

**DOI:** 10.1007/s00348-025-04118-6

**Published:** 2025-11-14

**Authors:** Subhajit Biswas, Paul Hayden, Matteo Carpentieri, Christina Vanderwel

**Affiliations:** 1 Department of Aeronautics and Astronautics, University of Southampton, Southampton, UK; 2 Environmental Flow (EnFlo) Laboratory, University of Surrey, Guildford, UK

## Abstract

In urban environments, pollutant ingress from outdoor sources poses a significant challenge to indoor air quality. Cross-ventilation, while essential for passive cooling and natural airflow, can also facilitate the entry of outdoor contaminants into indoor spaces. To investigate the dynamics of outdoor-to-indoor pollutant transport, the present study employs an idealized configuration, namely, a hollow cube representing a scaled-down model building with window openings in the upstream and downstream faces, subjected to an upstream passive scalar source within an atmospheric boundary layer. The experiments are conducted in two distinct facilities: a water tunnel using Rhodamine dye as the scalar, and a wind tunnel using propane gas, all performed at a specified flow Reynolds number of $\text{Re}  = U_{{{\text{Ref}}}} H/\nu  \approx 50,000$ for a fixed boundary layer-to-cube height ratio of about 3; here, $U_{{{\text{Ref}}}}$ is the streamwise velocity at cube’s height (*H*) measured without the cube. The scalar, released from a ground-level upstream source, is predominantly transported by a streamwise advective flux, while relatively weaker wall-normal advective and turbulent fluxes contribute to vertical dispersion and local mixing. A fraction of the oncoming scalar enters the cube intermittently, through the upstream window. Inside, a central jet-like flow carries the scalar parcels primarily by streamwise advective flux, while also interacting with the upper and lower recirculation regions, enabling scalar exchange across these zones through wall-normal advective and turbulent fluxes. While the time-averaged concentration field inside the cube is nearly uniform, suggesting effective mixing, instantaneous concentration traces exhibit strong intermittency, with sporadic peak events, highlighting the risk of transient peak exposures. The indoor concentration exponentially decays over time once the source is turned off, with a slower decay in the upper recirculation region, implying relatively prolonged exposure near the ceiling region. Both experimental setups produce closely matching values and consistent trends in the spatio-temporal dynamics of scalar concentration, and also highlight their complementary nature, with each offering distinct advantages. The present findings will deepen our understanding of pollutant ingress and mixing in buildings in cross-ventilated flows and also offer valuable insights to future modeling of pollutant exposure in urban indoor spaces.

## Introduction

Air pollution is a significant global public health threat, causing millions of deaths annually (World Health Organization 2019). In addition to its direct impact on morbidity and mortality, it also plays a crucial role in climate change, escalating environmental challenges worldwide (Manisalidis et al. 2020). Short-term exposure to air pollutants is linked to respiratory issues such as Chronic Obstructive Pulmonary Disease (COPD), shortness of breath, wheezing, and asthma, while long-term exposure has been associated with more severe conditions, including pulmonary insufficiency, cardiovascular diseases, and various types of cancer (Eze et al. 2014).

In urban environments, pollutants originate from both indoor and outdoor sources, contributing to deteriorating air quality. There has been an extensive body of research on the flow dynamics and pollutant dispersion in both indoor and outdoor environments (Jiang et al. 2023; Hanna 2003; Carpentieri 2013). Since humans spend most of their time (85–90%) indoors, evaluating indoor pollutant exposure is crucial for understanding its health implications. A number of previous studies have focused on modeling indoor pollutant dispersion, particularly in relation to flow patterns and pollutant spread (Blocken et al. 2013; Holmberg and Li 1998; Van Hooff et al. 2012; Zhang and Chen 2006; Lim et al. 2024). For example, studies have examined pollutant exchange between indoor spaces driven by natural ventilation (Ai and Mak 2015; Liu and Zhai 2007), as well as tracer gas dispersion in greenhouses and livestock buildings (Norton et al. 2009; Bartzanas et al. 2004).

Natural ventilation has a critical role in maintaining indoor air quality. It has been shown to prevent airborne contagion, mitigate the risks associated with Sick Building Syndrome, and enhance thermal comfort (Jiang et al. 2023; Escombe et al. 2007; Dutton et al. 2013; Lipczynska et al. 2018). To better understand these, research on indoor–outdoor air quality modeling has emphasized the critical role of ventilation in maintaining a sustainable and healthy indoor environment (Van Hooff and Blocken 2010; Tominaga and Stathopoulos 2013; Zhang and Chen 2009; Tong et al. 2016; Yang et al. 2015). Various studies have reported on the key mechanisms of ventilation using wind and water tunnel experiments, analytical models, and computational simulations (Kato et al. 1992; Biswas and Vanderwel 2024; Li and Delsante 2001; Van Hooff and Blocken 2010). For example, Yaghoubi et al. (1998) investigated turbulence distributions in and around cross-ventilated buildings, while Cheung and Liu (2011) examined the relationship between indoor and outdoor air quality in an isolated building using Large Eddy Simulation (LES).

Experimental studies have provided valuable insights by helping visualize pollutant transport in cross-ventilation scenarios. Tominaga and Blocken (2016) conducted wind tunnel experiments on cross-ventilating flow through a hollow cube (with indoor pollutants) having openings on opposite facades, investigating the impact of window placement on pollutant concentration and flushing efficiency. Their measurements significantly advanced the understanding of cross-ventilation transport mechanisms. Building on this, Kosutova et al. (2019) combined wind tunnel experiments and CFD simulations to analyze cross-ventilation in a similar model, incorporating louvers on windows. Their findings showed that window positioning significantly influences airflow patterns, air age, and exchange efficiency, with the presence of louvers further altering these parameters. More recently, Biswas and Vanderwel (2024) performed water tunnel measurements to investigate pollutant transport mechanisms in a flow through a hollow cube with an indoor pollutant source. Their simultaneous flow and pollutant concentration measurements highlighted how changing window positions strongly impacts pollutant transport mechanisms and mixedness, thus affecting the mean concentrations and their characteristic time scales. These studies collectively underscore the complex interplay between flow patterns, pollutant dispersion, and indoor air quality, offering key insights for optimizing natural ventilation in buildings.

The study of the interaction between indoor and outdoor air quality is crucial, as indoor pollutants originate not only from internal sources but are also significantly carried into the indoor environment from outdoor pollution sources due to the air exchange between the two environments (González-Martín et al. 2021; Yocom 1982). For example, with rising traffic and industrial emissions, more outdoor pollutants infiltrate indoor spaces, thus making it essential to understand how particulate matter is transported from outdoor sources into indoor environments (Blondeau et al. 2005; Hänninen and Goodman 2019). A number of researchers studied the impact of outdoor pollutants on indoor air quality, as these are transported via infiltration, natural ventilation, and mechanical ventilation (Park et al. 2014; Kulmala et al. 1999). Only a limited number of studies have integrated both indoor and outdoor environments into a single model, primarily due to the complexity and challenges involved (Mohammadi and Calautit 2021). For example, Tong et al. (2016) examined the pollution levels within an office space based on its proximity to a pollutant source. Yang et al. (2015) investigated how the percentage of window openings along a façade affects indoor air quality in a downstream building. Rather than directly measuring indoor pollutant levels, the study estimated ventilation flux to assess the indoor air quality. In typical indoor–outdoor exchange scenarios, the extent to which outdoor traffic pollution affects indoor spaces is governed by several factors, including building layout, street canyon geometry, ventilation flow rates, and wind direction (He et al. 2017; Tominaga and Stathopoulos 2016). While many of these studies primarily focused on ventilation efficiency and indoor–outdoor pollutant concentration ratios (Brunekreef et al. 2005; Meng et al. 2005), a significant gap still remains in understanding the flow dynamics, the pollutant transport mechanisms, and their stochastic characteristics for ventilating flows with outdoor pollutant sources.

**Fig. 1:**
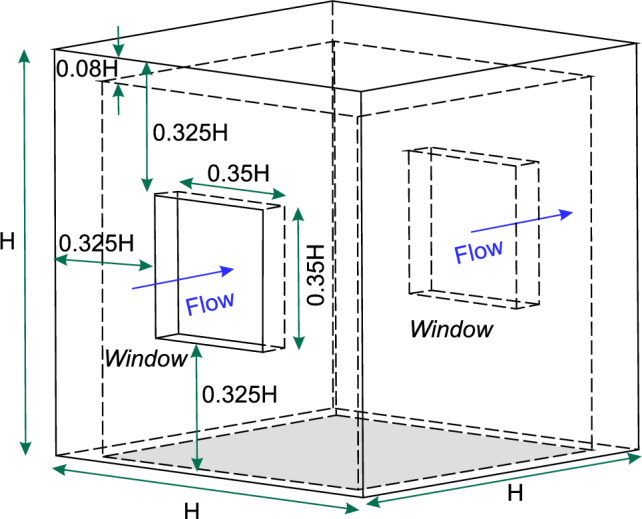
Schematics showing a 3D view of the hollow building model used in this study, with flow direction from left to right. All dimensions are scaled relative to the cube height (*H*)

**Fig. 2:**
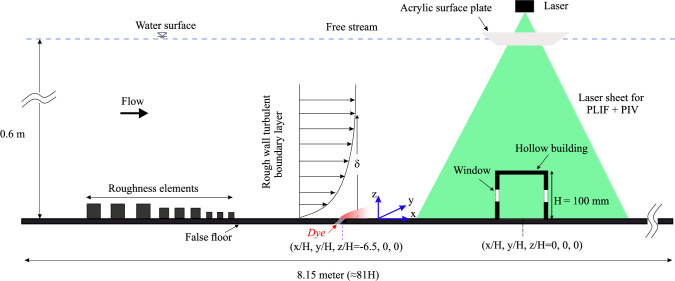
This schematic illustrates a side view of the experimental setup in the water tunnel facility. The hollow cube model was mounted on a false floor panel atop the glass floor of the flume’s test section (blockage ratio $<1\%$). Simultaneous measurements using planar laser-induced fluorescence (PLIF) and particle image velocimetry (PIV) were conducted in the streamwise plane ($x - z$) passing through both the building centerline ($y = 0$, spanwise) and dye source, capturing both scalar concentration and velocity fields

To bridge this gap, the present study experimentally investigates pollutant transport mechanisms in a cross-ventilating flow through a scaled-down hollow cubic building featuring upstream and downstream windows (see Fig. 1). The building model is placed within a rough-wall turbulent boundary layer flow, with a ground-level passive scalar pollutant source located far upstream (see Fig. 2). The present work builds upon our recently reported study (Biswas and Vanderwel 2024) on flow and dispersion in a similar configuration with an indoor source. In contrast, the present focus has been on understanding the transport of scalar originating from an outdoor source placed upstream. Experiments are performed in the water tunnel facility at University of Southampton and the *Enflo* wind tunnel facility at University of Surrey. To our knowledge, this study provides the first detailed analysis of simultaneous flow and dispersion measurements in a cross-ventilated flow setup with an outdoor source. It is also worth noting that, to date, most experimental studies on flow and pollutant dispersion have been conducted primarily in wind tunnel facilities, with the use of water tunnels being comparatively rare. While the primary objective of the present study is to investigate indoor dispersion with an outdoor source, our approach also highlights the comparison of the measurements across these experimental setups, and how these act in a complementary manner, with each offering distinct advantages. The change in source location fundamentally alters the scalar influx mechanism, thereby modifying the scalar transport processes and their spatio-temporal dynamics as compared with Biswas and Vanderwel (2024). The structure of the article is as follows. The experimental methodologies are described in §2, followed by §3 providing insights into the flow dynamics and scalar transport. Finally, §4 provides a comprehensive summary and conclusions.

## Experimental methods

### Building model

The simplified building was modeled as a hollow acrylic cube with height *H* as pictured in Fig. 1. The building had two opposite openings, each being $0.35H \times 0.35H$, on the windward and leeward facades, yielding an area-based porosity of about 10%, consistent with Biswas and Vanderwel (2024).

**Fig. 3:**
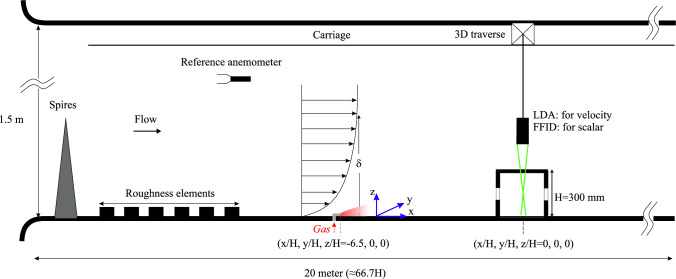
Schematic showing the side view of the experimental arrangements used in the wind tunnel facility. A scalar (propane gas) injection from the building’s floor was facilitated at 6.5*H* upstream. On the measurements side, FFID and LDA measurements were performed at different points, as described in Fig. 5, to capture the scalar concentration and velocity fields, respectively

Analogous experiments were conducted in a water tunnel facility (see Fig. 2) and a wind tunnel facility (see Fig. 3) with parameters as summarized in Table 1. In the water tunnel, a hollow cubic model with an exterior height, *H*, of 100 mm ($\approx$ 1:40 scale to full size, as in Biswas and Vanderwel (2024)) was used with a reference water speed, $U_{{{\text{Ref}}}}$, of 0.45 m/s at the cube height measured without the cube, yielding a Reynolds number of $\text{Re}  = U_{{{\text{Ref}}}} H/\nu  \approx 50,000$. The wind tunnel experiment used a larger cube with a height of 300 mm ($\approx$ 1:13 scale) and a reference wind speed of 2.5 m/s at the cube height, achieving a Reynolds number of $\approx 50,000$, equivalent to that in the water tunnel. In both setups, the boundary layer-to-building height ratio, ${\delta }/H$, was about 3. It is important to note that for the normalization of data from the two facilities, the respective cube heights and reference velocities were used.

**Table 1:** Summary of different parameters from both the facilities, including cube’s height (*H*), streamwise reference speed of the oncoming flow at cube’s height ($U_{{{\text{Ref}}}}$) measured without the cube, boundary layer thickness ($\delta$), boundary layer-to-cube height ratio ($\delta /H$), and Reynolds number based on the reference flow speed and cube height ($\text{Re}  = U_{{{\text{Ref}}}} H/\nu$, $\nu =$ kinematic viscosity of the fluid medium). It is important to note that for the normalization of data from the two facilities, the respective cube heights and reference velocities were used

Facility	*H* (mm)	$U_{{{\text{Ref}}}}$ (m/s)	$\delta$ (mm)	$\delta /H$	$\text{Re} = U_{{{\text{Ref}}}} H/\nu$
Water tunnel	100	0.45	310	3.0	$\approx 50,000$
Wind tunnel	300	2.5	930	3.1	$\approx 50,000$

### Water tunnel setup

The recirculating water tunnel facility at the University of Southampton features a test section measuring 8.1 m in length, 1.2 m in width, and 0.9 m in height. The facility has three walls, the bottom wall and two transparent side walls, and an open top. The hollow cube was mounted on a false floor positioned over the glass floor of the test section. Upstream of the test section, a series of roughness blocks of varying sizes were used to generate a fully developed rough-wall turbulent boundary layer downstream in the test section, simulating atmospheric boundary layer conditions, similar to those described in our previous studies (Lim et al. 2022; Rich and Vanderwel 2024; Biswas and Vanderwel 2024).

The two-dimensional velocity and dye concentration fields within and around the hollow cube were captured using simultaneous particle image velocimetry (PIV) and planar laser-induced fluorescence (PLIF) measurements taken in the streamwise plane (*x*−*z*) along the building’s centerline (spanwise, $y = 0$). This measurement plane was also aligned with the ground-level pollutant source placed far upstream to the hollow cube model, at a distance of 6.5*H* measured from the cube’s center, as illustrated in Fig. 2. The streamwise field of view spanned from ‘$-1.5H$’ to ‘1.5*H*’ (from cube’s center, $x = 0$), and ‘2*H*’ in the wall-normal direction (in *z*). The flow was illuminated by a 100 mJ Nd double-pulsed laser emitting light at a wavelength of 532 nm. The experimental setup used two cameras equipped with specialized filters to capture the PIV and PLIF signals. The velocity and concentration fields were captured simultaneously at a rate of 10 Hz (see our previous studies (Lim et al. 2022; Biswas and Vanderwel 2024) for additional details on the experimental arrangements and PIV processing).

The injection of a neutrally buoyant aqueous solution of Rhodamine 6G fluorescent dye was embedded in the false floor upstream to the cube, simulating a ground-level source of a passive scalar pollutant, with negligible influence on the surrounding flow (similar to Lim and Vanderwel (2023)). The dye solution, with a concentration ($C_S$) of 100 mg/L, was delivered at a constant flow rate ($\dot{V}$) of $1.67 \times 10^{-4}$ liter/s using a needle valve and a Mariotte bottle. The dye solution was injected via a thin tube connected to an internal channel in the acrylic false floor, which directed the solution through a 5 mm diameter hole angled at 45$^o$ to the floor (see Fig. 2). The injection velocity was approximately 8 mm/s, and its ratio to $U_{{{\text{Ref}}}}$ was 0.017, indicating that the injection had negligible influence on the flow. The dye concentration measurements (*C*, in mg/liter) are normalized as,

1 $$\begin{aligned} C^{*} = \frac{CA U_{\text {Ref}}}{Q_S} \end{aligned}$$

where, $Q_S=C_S$ $\dot{V}$, is the rate of scalar injection (in mg/s) at the source, $\dot{V}$ is the rate of injection of the aqueous solution of dye (in liter/s), and $C_S$ is the dye concentration in the solution (mg/liter) and $A=H^2$. This normalization accounts for flow speed and building size, and is consistent with the previous studies on dispersion in urban environments (e.g., Robins (1980); Snyder (1981); Xia et al. (2014); Daniela et al. (2012); Tominaga and Blocken (2016)). This ensures a fair comparison between water and wind tunnel measurements, as will be demonstrated later. The dye’s Schmidt number (*Sc*) was approximately $2500\pm 300$ (Vanderwel and Tavoularis 2014), implying that momentum diffused much faster than the scalar. The water quality in the flume was continuously monitored, where overnight UV sterilization prevented microbial growth, while mechanical filtration removed fine suspended particles and debris. This combination ensured clean water for Particle Image Velocimetry (PIV) and Planar Laser-Induced Fluorescence (PLIF) measurements. In addition, the water quality was also monitored to avoid residual dye accumulation, which remained minimal due to the large tank volume ($\sim$30,000 L) and the overnight chlorine treatment that broke down any dye build-up, thereby maintaining the reliability and accuracy of the PLIF measurements. The dye was released from a ground-level source upstream of the cube at a distance (in *x*) of 6.5*H* from the center of the building, and this distance was sufficient for the plume to have dispersed a moderate amount in the boundary layer before reaching the cube. The local scalar (dye) concentration was quantified based on fluorescence intensity, following a methodology discussed in our previous studies (Lim et al. (2022); Vanderwel and Tavoularis (2014)). Since the velocity and concentration fields were captured (simultaneously) in different co-ordinate systems at distinct spatial resolutions, these were mapped onto a unified co-ordinate system, enabling the computation of joint velocity–concentration statistics. A bootstrap method was applied to the first- and second-order statistics of both the PIV and PLIF measurements. For PIV, the standard error in the mean streamwise velocity was determined to be less than 1%, while it was under 5% for the variance. For PLIF, the standard error in the mean concentration was under 1% and 3% in variance. The results showed that 1000 samples were adequate for achieving converged statistics. The uncertainty associated with the joint statistics was below 5%, ensuring reliable and accurate results.

### Wind tunnel setup

A set of experiments similar to the water tunnel study was carried out in the *EnFlo* wind tunnel facility at the University of Surrey, employing laser Doppler velocimetry (LDA) for velocity measurements and fast-response flame ionization detector (FFID) concentration measurements. This facility is an open-circuit suction-type boundary-layer wind tunnel, featuring a large working section measuring 20 m $\times$ 3.5 m $\times$ 1.5 m (length $\times$ width $\times$ height). To replicate atmospheric boundary layer conditions, a set of Irwin spires was positioned 0.5 m downstream of the test section inlet (as in Marucci et al. (2018)). Each spire is 1260 mm tall, with a base width of 170 mm and a tip width of 4 mm. The spires were spaced laterally at intervals of 630 mm. Downstream of the spires, a staggered array of roughness elements was placed on the wind tunnel floor extending up to the gas injection location, as illustrated in Fig. 3. These roughness elements are 20 mm tall and 80 mm wide, with lateral and longitudinal spacings of approximately 240 mm between individual elements. A turntable with a diameter of 1.5 m was located 14 m downstream of the inlet, serving as the mounting platform for the hollow cube model (height, *H*=300 mm). The cube was mounted such that its ground-level center aligned precisely with the center of the turntable. The blockage ratio was less than $1.5\%$. For consistency, the origin of the co-ordinate system was defined at the ground-level center of the cube, as illustrated in Fig. 3, similar to the water tunnel study.

Pointwise velocity measurements were conducted using a laser Doppler anemometry (LDA) system (FiberFlow, Dantec Dynamics) to simultaneously measure the streamwise (*U*, along *x*) and spanwise (*V*, along *y*) velocity components, due to experimental constraints. For the base flow without cube, the streamwise (*U*) and wall-normal (*W*, along *z*) velocity components were measured. The LDA rig was mounted on a traverse capable of three-dimensional independent movement along the *x*, *y* and *z* axes. An aerosol solution of sugar particles with a mean diameter of approximately 1$\mu$m was employed as tracers, generated using an in-house ultrasonic mist generator. The target acquisition frequency was set to 100 Hz, with a sampling duration of 2 minutes for each measurement, and was found to be sufficient to achieve statistical convergence. Statistical errors were found to be within ±0.5% for the mean velocity and ±5% for the second-order moments, with a 95% confidence level.

A circular source with a diameter of 22 mm was positioned at ground level, at the spanwise center ($y=0$) of the wind tunnel, and located 6.5*H* upstream (in ‘*x*’) from the center of the cube, as shown in Fig. 3. The tracer gas consisted of a mixture of propane (at a concentration of less than 1.5%) in air and had an exit velocity maintained at approximately $0.017U_{{{\text{Ref}}}}$ to ensure passive injection, similar to water tunnel measurements. The tracer was sufficiently diluted to eliminate buoyancy effects. The relatively large diameter of the source, combined with a very low injection flow rate minimized momentum effects associated with the release, leaving residual effects that were negligible except in the immediate vicinity of the release location. The concentration data (*C*) was recorded as a time series using FFID system, measuring hydrocarbon concentrations at a frequency of 200 Hz, following the methods described by Auerswald et al. (2024). The building model was made with a set of small holes (about 4 mm in diameter) on the top surface, allowing the FFID probe to traverse into the cube for indoor concentration measurements. For LDA measurements, the laser beam passed through these holes to perform indoor velocity measurements. Given the small size of the holes, their effect on the internal flow and concentration fields was determined to be negligible. This was validated by comparing test results of concentration measurements with the holes open and with the holes sealed; the results were found to be identical. The gas concentration measurements (*C*, in ppm) are normalized as,

2 $$\begin{aligned} C^{*} = \frac{CA U_{\text {Ref}}}{Q_S} \end{aligned}$$

where $A=H^2$, and $Q_S$, is the flow rate of pure propane at the source, as was described by Marucci and Carpentieri (2020). The Schmidt number for propane gas is $Sc\approx 1$, much smaller compared to that of Rhodamine dye in water ($Sc \approx 2500\pm 300$). However, as we will see later, the spatio-temporal characteristics of the normalized concentration are remarkably similar across both facilities. The wind tunnel measurements, including the LDA, FFID, and traverse movements, were fully automated and co-ordinated using custom in-house software developed in LabVIEW.

**Fig. 4:**
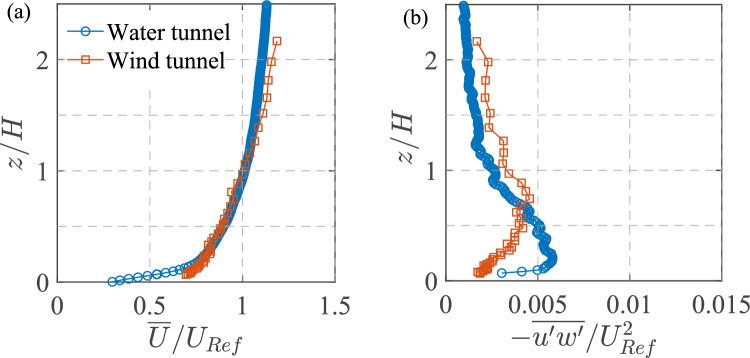
Characterization of the incoming flow at *Re* of 50,000, in terms of the wall-normal (*z*/*H*; *H*=cube height) profiles of the normalized:** a** mean streamwise velocity ($\overline{U}/U_{{{\text{Ref}}}}$), and** b** Reynolds stress ($-\overline{u^{\prime }w^{\prime }}/U^2_{\text{Ref}}$). These measurements were taken in the water tunnel ( 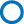, blue circles) and wind tunnel (, orange squares) test sections (at $x/H, y/H=-1.5, 0$), without the cube model

### Boundary layer characterization

Before beginning experiments involving the cube, the incoming flow in the two different setups was characterized in terms of the boundary layer profiles without the model in the test section. In Fig. 4, the wall-normal (*z*/*H*) profiles of the mean streamwise velocity ($\overline{U}/U_{{{\text{Ref}}}}$) and the Reynolds stress ($-\overline{u^{\prime }w^{\prime }}$/$U^2_{\text{Ref}}$) are shown; here, $\overline{U}$= mean (time-averaged) streamwise velocity, $u^{\prime }$= streamwise fluctuating velocity, and $w^{\prime }$= wall-normal fluctuating velocity. In Fig. 4a, $\overline{U}/U_{{{\text{Ref}}}}$ for the wind tunnel and water tunnel show substantial similarity. It may be noted that even though the boundary layer thickness ($\delta$) values in both the facilities were different (see Table 1), its ratio to the cube’s height was kept constant about 3, and the oncoming flow Reynolds number (*Re*) was fixed at $\approx 50,000$. These demonstrate the consistency between the two setups despite differences in fluid properties, such as density and viscosity. The close overlap of the two profiles also suggests that both experiments successfully replicate the canonical behavior of an atmospheric turbulent boundary layer when normalized using appropriate scaling parameters, such as $U_{\text {Ref}}$ and *H*. Subtle differences can be observed in the near-wall region ($z/H < 0.2$), where the wind tunnel data show slightly higher velocity values compared to the water tunnel. This discrepancy could be attributed to some differences in the experimental setup, such as the surface roughness.

Figure 4b shows the turbulence characteristics by comparing the Reynolds stress ($-\overline{u'w'}/U_{\text {Ref}}^2$) profiles in the wind and water tunnels. As can be seen from both datasets, while the overall shape and order of magnitude of the Reynolds stress profiles are consistent between the two setups, the values differ slightly. The wind tunnel data exhibit slightly lower Reynolds stress values near the wall, indicating lesser turbulence production than the water tunnel. Despite some minor differences, the overall consistency, both qualitative and quantitative, in the general trends of mean velocity and Reynolds stress profiles highlights the similarity in boundary layer inflow conditions between the two experimental facilities employing different fluid media.

**Fig. 5:**
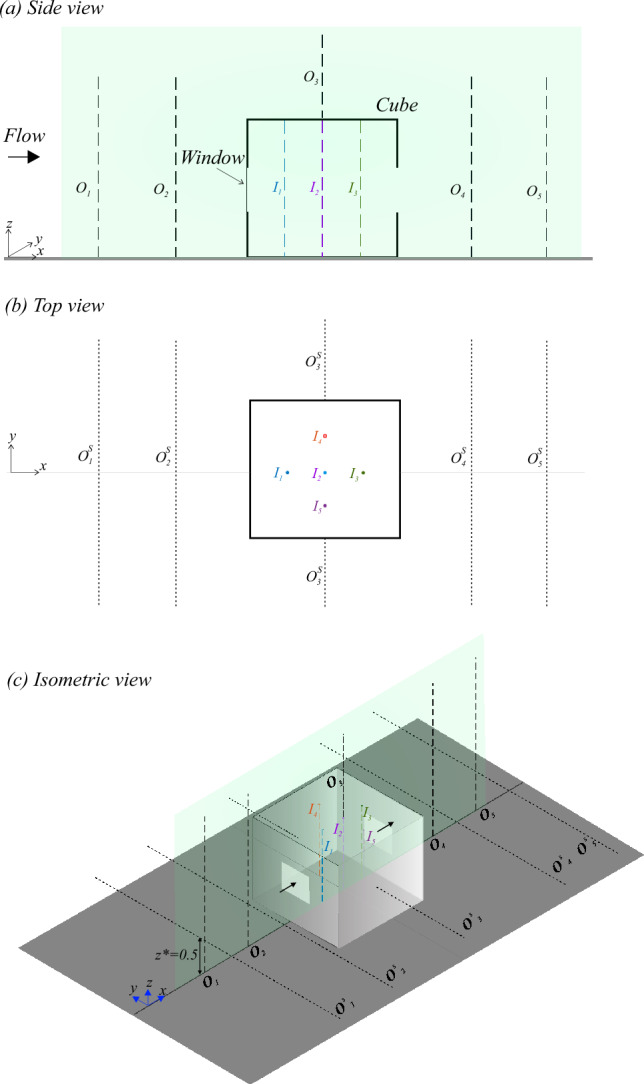
We have shown here the **a** side view of the center plane, **b** top view, and **c** isometric view of the experimental configuration, to illustrate the measurement locations in the water tunnel and wind tunnel. In the water tunnel, PIV and PLIF measurements were conducted both inside and outside the cube in the spanwise center plane ($y=0$), as indicated by the green transparent plane. In the wind tunnel, LDA and FFID measurements were taken along different lines at various streamwise locations (*x*). The wall-normal (along *z*) profiles were measured at $O_1$, $O_2$, $O_3$, $O_4$, and $O_5$ (outdoor), as well as at $I_1$, $I_2$, $I_3$, and $I_4$ (indoor). Additionally, (outdoor) spanwise profiles ($O^{S}_1$, $O^{S}_2$, $O^{S}_3$, $O^{S}_4$, $O^{S}_5$: All along *y*) were measured at those streamwise locations as in $O_1$ to $O_5$, all at a fixed wall-normal height of $z^{*}=z/H = 0.5$. The details of these measurement locations for each of these wall-normal and spanwise profiles are given in Table 2

### Measurement locations

To analyze scalar dispersion, measurements of scalar concentration and velocity were taken at various locations, including the cube’s indoor regions, and also upstream, above, and downstream of the cube, as illustrated in Fig. 5. In the water tunnel, simultaneous PIV and PLIF measurements were conducted in a streamwise plane covering a field of view of approximately 3*H* in the streamwise direction (in ‘*x*’) and 2*H* in the wall-normal direction (‘*z*’). This measurement plane passed through the ground-level center of the cube ($x^{*}, y^{*}, z^{*}=0,0,0$) and was aligned with the scalar source placed upstream at ($x^{*}, y^{*}, z^{*}=-6.5,0,0$); here, the co-ordinate axes were normalized by the cube height *H*, as $x^{*}=x/H$, $y^{*}=y/H$, and $z^{*}=z/H$. In the wind tunnel, velocity and gas concentration were measured along the wall-normal (*z*) and spanwise (*y*) directions at various streamwise locations (*x*), using LDA and FFID, respectively, as indicated by the dashed lines in Fig. 5. The measurement locations are categorized into three groups: wall-normal (*z*) and spanwise (*y*) profiles outside the cube, and wall-normal (*z*) profiles inside the cube, as summarized in Table 2. These measurement locations are now further elaborated below.

The outdoor measurements along the wall-normal direction ($z^{*}$) were conducted at five streamwise locations, denoted as $O_1$ to $O_5$. These positions are indicated by black dashed lines (----) in Fig. 5. Upstream of the cube, the measurement locations are at the spanwise center, $y^{*}=0$, and are as follows: $O_1$ at $x^{*} = -1.5$, and $O_2$ at $x^{*} = -1$. Above the cube, $O_3$ is positioned at $x^{*} = 0$, and downstream of the cube $O_4$ is located at $x^{*} = 1$ and $O_5$ at $x^{*} = 1.5$. In addition to the wall-normal profiles, also measured were the spanwise ($y^{*}$) profiles conducted along the black dotted lines (........) at a fixed wall-normal height of $z^{*} = 0.5$. These spanwise lines are denoted as $O^S_1$ to $O^S_5$. Upstream of the cube, the positions of these lines are at $O^S_1$ ($x^{*} = -1.5$) and $O^S_2$ ($x^{*} = -1$). Spanwise measurements on either side of the cube were taken at $O^S_3$ ($x^{*} = 0$). Further downstream, additional measurements were conducted at $O^S_4$ ($x^{*} = 1$) and $O^S_5$ ($x^{*} = 1.5$).

Inside the cube, wall-normal profiles for velocity and concentration were obtained along five vertical lines, denoted as $I_1$ to $I_5$. These measurements provide insights into the internal flow structure and indoor scalar distribution. The wall-normal lines in the center plane (at $y^{*} = 0$) are as follows: $I_1$ at $x^{*} = -0.25$, $I_2$ at $x^{*} = 0$, and $I_3$ at $x^{*} = 0.25$. Additionally, wall-normal profiles at the cube’s streamwise center ($x^{*} = 0$) were taken at $I_4$ ($y^{*} = 0.25$) and $I_5$ ($y^{*} = -0.25$), situated on either side (spanwise) of the center plane. As we will discuss later, these two wall-normal profiles ($I_4$ and $I_5$) help to better understand the three-dimensional (volumetric) variations of the scalar concentration.

It is noteworthy that simultaneous PLIF and PIV measurements in the water tunnel captured two-dimensional fields of both velocity and scalar concentrations, as well as scalar fluxes through their product. However, these measurements were limited to the spanwise center plane due to experimental constraints, which prevented access to other spanwise and wall-parallel planes. In contrast, while simultaneous FFID and LDA measurements were not feasible in the wind tunnel owing to experimental constraints, measurements across various spanwise and wall-normal planes were readily achievable. Thus, the two experimental facilities and measurement techniques complemented each other, with each offering distinct advantages that together provided a more comprehensive dataset.

**Table 2:** Summary of the locations of the lines (as shown in Fig. 5) along which wall-normal ($z^{*}$) and spanwise ($y^{*}$) profiles of velocity and concentration were measured in the wind tunnel facility. Also shown is the laser light sheet in the streamwise plane passing through the center of the cube ($x,y,z=0,0,0$), as in the water tunnel

Setup	Profile	Notation	$x^{*}$	$y^{*}$ & $z^{*}$
Wind tunnel	Wall-normal (Upstream)	$O_1$	-1.5	$y^{*} = 0$
	– (Upstream)	$O_2$	-1	$y^{*} = 0$
	– (Rooftop)	$O_3$	0	$y^{*} = 0$
	– (Downstream)	$O_4$	1	$y^{*} = 0$
	– (Downstream)	$O_5$	1.5	$y^{*} = 0$
	Spanwise (Upstream)	$O^S_1$	-1.5	$z^{*} = 0.5$
	– (Upstream)	$O^S_2$	-1	$z^{*} = 0.5$
	– (Either sides)	$O^S_3$	0	$z^{*} = 0.5$
	– (Downstream)	$O^S_4$	1	$z^{*} = 0.5$
	– (Downstream)	$O^S_5$	1.5	$z^{*} = 0.5$
		$I_1$	-0.25	$y^{*}=0$
		$$ I_2$$	0	$$ y^{*}=0$$
	Wall-normal (Indoor)	$I_3$	0.25	$y^{*}=0$
		$I_4$	0	$y^{*}=0.25$
		$I_5$	0	$y^{*}=-0.25$
Water tunnel	PLIF and PIV	Laser sheet	-1.5 to 1.5	$y^{*} = 0, z^{*} = 0-2$

**Fig. 6:**
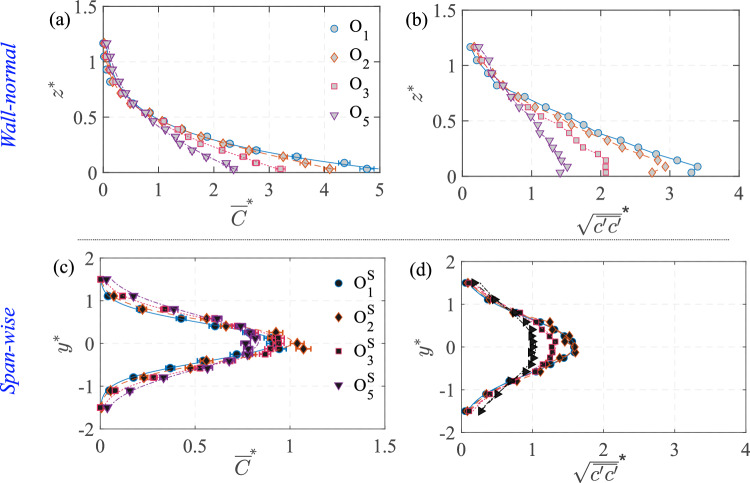
Characterization of the scalar field (without cube) in the wind tunnel test section at different streamwise locations, is shown here, in terms of the wall-normal ($z^{*}=z/H$) and spanwise ($y^{*}=y/H$) profiles of the mean (time-averaged) concentration (${\overline{C}}^{*}=\overline{C}AU_{{{\text{Ref}}}}/Q_S$) and its variance (${\sqrt{\overline{{c^{\prime }}^2}}}^{*}={\sqrt{\overline{{c^{\prime }}^2}}}AU_{{{\text{Ref}}}}/Q_S$). The streamwise locations of $O_1$, $O_2$, $O_3$, and $O_5$ are shown in Table 2

**Fig. 7:**
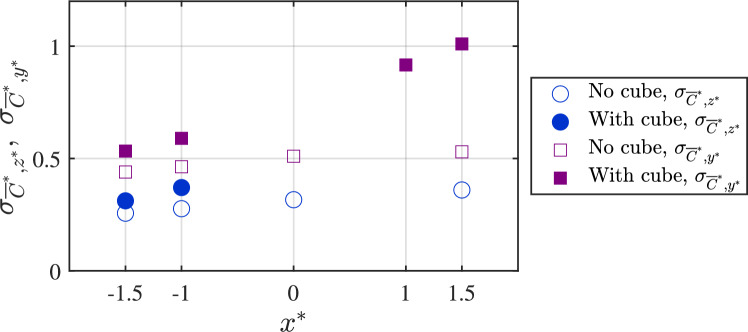
The vertical and horizontal spread of the scalar plume in the wind tunnel, in terms of $\sigma _{{\overline{C}}^{*}, z^{*}}$ and $\sigma _{{\overline{C}}^{*}, y^{*}}$, respectively, are shown at different streamwise locations ($x^{*}$). These are obtained from Gaussian fit, as in Equations 3 & 4. These measurements are shown for both the base case (no-cube) and with the cube. It may be noted that $\sigma _{{\overline{C}}^{*}, z^{*}}$ are not obtained for ‘
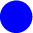
’ from $x^{*}=0$ onward since the plume no longer followed a Gaussian distribution due to the presence of the cube

### Scalar field characterization without cube

The analysis of scalar dispersion begins with the measurement of the concentration and variance of the scalar plume without the cube. Figure 6a,c provides the mean (time-averaged) scalar (${\overline{C}}^{*}=\overline{C}AU_{{{\text{Ref}}}}/Q_S$) distribution, while the concentration variance (${\sqrt{\overline{c^{\prime }c^{\prime }}}}^{*}=\sqrt{\overline{c^{\prime }c^{\prime }}}AU_{{{\text{Ref}}}}/Q_S$) in Fig. 6(b,d) quantifies the intensity of concentration fluctuations; here, $c^{\prime }$=*C*-$\overline{C}$, where *C* is the instantaneous concentration, and $\overline{C}$ is the time-averaged concentration. In Fig. 6a, profiles are shown along the wall-normal direction ($z^{*}$) at four streamwise locations at $y^{*}=0$: $O_1 \quad (x^{*} = -1.5)$, $O_2 \quad (x^{*} = -1)$, $O_3 \quad (x^{*} = 0)$, and $O_5 \quad (x^{*} = 1.5)$. The profiles in Fig. 6a follow one half of a Gaussian distribution, showing that the scalar concentration peaks near the wall and gradually diffuses upward, as,

3 $$\begin{aligned} {\overline{C}}^{*}(z^{*}) = A \exp \left( -\left( \frac{z^{*} - \mu _{{z^{*}}}}{\sqrt{2} \sigma _{{\overline{C}}^{*},z^{*}}} \right) ^2 \right) \end{aligned}$$

where *A* represents the peak, $\mu _{{z^{*}}}$ represents the center of the Gaussian which is close to $z^*=0$, and $\sigma _{{\overline{C}}^{*},z^{*}}$ represents the half-width, determined through a least-squares fit similar to Marucci and Carpentieri (2020). At $O_1$, the near-ground concentration is highest, reflecting its proximity to the injection source. As the flow progresses downstream toward $O_5$, the concentration profiles flatten, suggesting increased turbulent mixing that redistributes the scalar (gas) across the boundary layer (in *z* and *y*). The other important quantity is the variance in Fig. 6b, representing the fluctuations in the concentration. The variance profiles indicate that scalar fluctuations are strongest near the wall due to higher turbulence closer to the surface, and then decrease with increasing $z^{*}$. At all streamwise locations ($O_1$ to $O_5$), the variance also follows a Gaussian distribution, similar to the mean concentration, though with a peak slightly above the ground. The variance decreases progressively downstream, with $O_1$ exhibiting the highest variance, indicating these fluctuations to be gradually attenuated as mixing progresses. The evolution of the plume half-width, $\sigma _{{\overline{C}}^{*},z^{*}}$, at different streamwise locations ($x^{*}$) is presented in Fig. 7 showing a steady widening of the plume downstream.

Figure 6c,d illustrates the variation of mean concentration and concentration variance along the spanwise direction ($y^{*}$) at a fixed height of $z^{*} = 0.5$. These measurements are taken at four streamwise locations, designated as $O^S_{1}$, $O^S_{2}$, $O^S_{3}$, and $O^S_{5}$, and these again correspond to the same streamwise positions for the wall-normal profiles in Fig. 6a,b. The mean concentration (${\overline{C}}^{*}$) profiles in Fig. 6c exhibit a Gaussian distribution centered at $y^{*} = 0$, characteristic of a canonical plume. As the flow progresses downstream, the concentration spreads laterally, as evidenced by a broadening of the profiles from $O^S_{1}$ to $O^S_{5}$, and also from Fig. 7. Following this, Fig. 6d presents the concentration variance (${\sqrt{\overline{c^{\prime }c^{\prime }}}}^{*}$) profiles along in $y^{*}$ at the wall-normal position of $z^{*} = 0.5$, showing a symmetric lateral profile. Similar to the wall-normal distributions, also notable is the increasing spanwise width of the plume in terms of $\sigma _{{\overline{C}}^{*},y^{*}}$ (shown in Fig. 7), obtained from fitting a Gaussian to the concentration profile as,

4 $$\begin{aligned} {\overline{C}}^{*}(y^{*}) = B \exp \left( -\left( \frac{y^{*} - \mu _{{y^{*}}}}{\sqrt{2} \sigma _{{\overline{C}}^{*},y^{*}}} \right) ^2 \right) \end{aligned}$$

where *B*, $\mu _{{y^{*}}}$, and $\sigma _{{\overline{C}}^{*},y^{*}}$ are the fitting parameters. Figure 7 presents the evolution of the plume half-widths. This plot shows that by the time, the plume reached the cube, the full plume width in the spanwise direction was on the order of the size of the cube ‘*H*.’

**Fig. 8:**
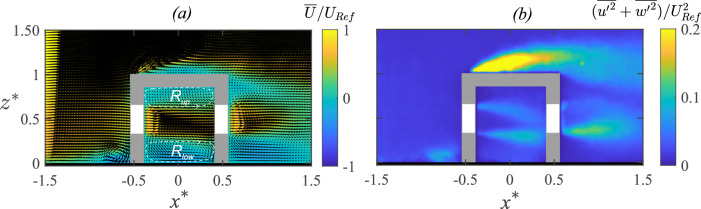
Time-averaged maps of the:** a** vector overlaid with streamwise velocity [$\overline{U}/U_{{{\text{Ref}}}}$] and** b** in-plane turbulent kinetic energy $[{(\overline{{u^{\prime }}^2}+\overline{{w^{\prime }}^2})/U^2_{\text{Ref}} }]$, all obtained from water tunnel measurements. The flow is from left to right. These measurements are performed in a streamwise plane ($x-z$ plane, at $y=0$) passing through the center of the hollow cube

**Fig. 9:**
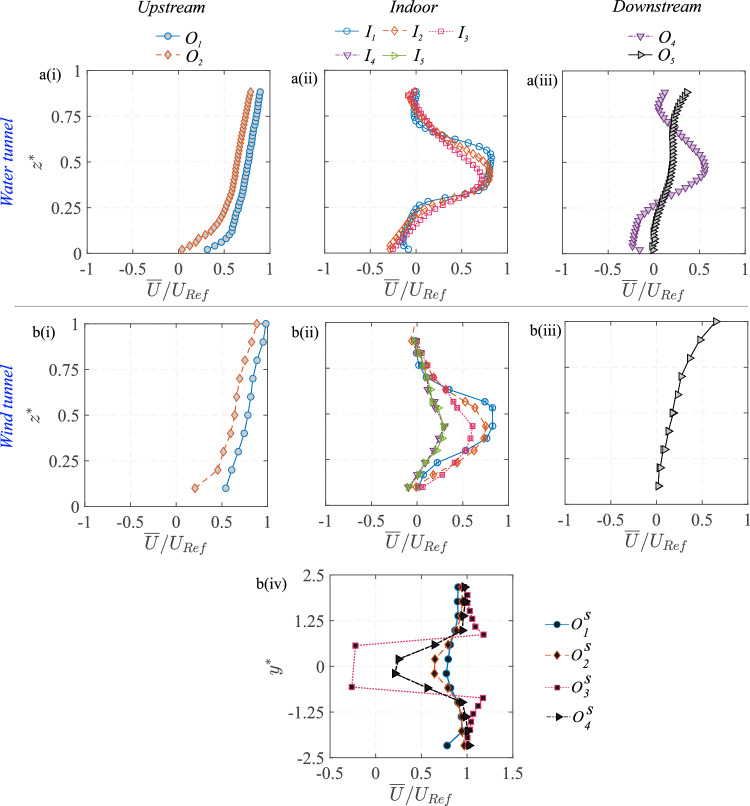
Measurements from the water tunnel and wind tunnel are shown for the wall-normal ($z^{*}$) profiles of the streamwise velocity ($\overline{U}/U_{{{\text{Ref}}}}$), measured upstream to the model [a(i), b(i)], inside [a(ii), b(ii)], and downstream [a(iii), b(iii)]. Also shown in ‘b(iv)’ are the spanwise ($y^{*}$) profiles of $\overline{U}/U_{{{\text{Ref}}}}$ from the wind tunnel, at different streamwise locations outdoors

## Results

A systematic analysis of the flow patterns, the scalar concentration distribution, and transport mechanisms is now presented in the presence of the hollow cube. These were captured from the wind and water tunnel measurements, employing distinct methodologies in each setup. The characterization of the flow field and scalar transport is presented at three regions: upstream of the cube, within the cube, and downstream of it.

### Velocity field

The flow through and around the hollow cube is illustrated in Fig. 8a showing the mean (time-averaged) streamwise velocity ($\overline{U}/U_{{{\text{Ref}}}}$) in the $x-z$ center plane (at $y^{*}=0$), as obtained using PIV in the water tunnel experiments. In addition, Fig. 9 shows the profiles of $\overline{U}/U_{{{\text{Ref}}}}$ along $z^{*}$ at different streamwise locations, obtained from both wind tunnel and water tunnel measurements. The oncoming flow approaching the cube undergoes deceleration, and this is evident in the velocity map in Fig. 8a and the wall-normal profiles upstream to the cube (at $O_1$ & $O_2$) in Fig. 9[a(i), b(i)]. Following these, a higher-magnitude streamwise velocity is seen above the cube (Fig. 8a), indicating flow acceleration on the rooftop. Also noticeable is the stagnation region and recirculation bubble near the ground that forms at the windward face of the cube. Now, coming to the indoor flow, as the flow passes through the cube, two recirculation regions are seen to form, evidenced by regions of reversed flow [$(-)ve \,\overline{U}$], indicated using white dashed closed lines in Fig. 8a. In between these regions, a jet-like flow moves through the center of the cube. This jet-like flow pattern is also visible in Fig. 9[a(ii), b(ii)] illustrating the wall-normal ($z^{*}$) profiles of the streamwise velocity at different indoor locations ($I_1$, $I_2$, and $I_3$). The instantaneous flow fields (not shown here) reveal that this jet is highly unsteady, exhibiting oscillations and interacting with the recirculation regions. Such interactions significantly influence mass and momentum exchange within the indoor environment. The broad flow characteristics are in line with the previous studies (e.g., Biswas and Vanderwel (2024); van Hooff et al. (2017); Perén et al. (2015)) reporting on cross-ventilating flows for generic building configurations.

**Fig. 10:**
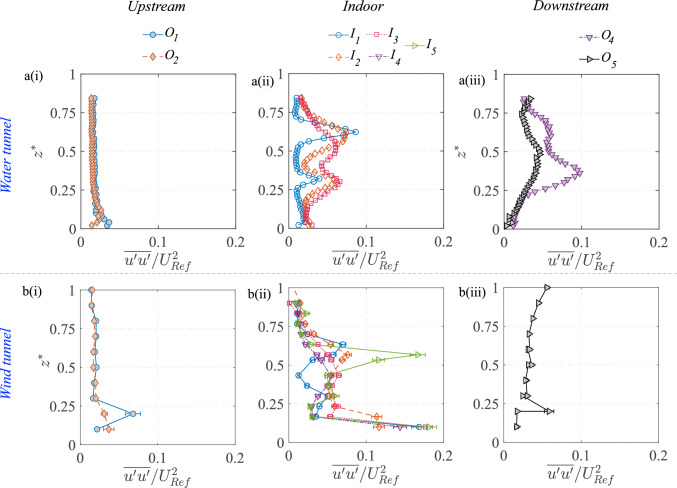
Measurements from the water tunnel and wind tunnel are shown for the wall-normal ($z^{*}$) profiles of the wall-normal component of (in-plane) TKE ($\overline{{u^{\prime }}^2}/U^2_{\text{Ref}}$), measured upstream to the model [a(i), b(i)], inside [a(ii), b(ii)], and downstream [a(iii), b(iii)]

It can be noted that both the water and wind tunnel measurements were performed in the spanwise center plane ($y^{*}=0$) while the wind tunnel also performed some wall-normal measurements at out-of-plane positions ($I_4$ at $y^{*}=0.25$, $I_5$ at $y^{*}=-0.25$), which indicate that the jet’s strength weakens in the spanwise direction away from the center plane. Downstream of the cube, Fig. 9[a(iii), b(iii)] depicts the wake flow structure measured at $O_4$ and $O_5$. The velocity profiles exhibit a significant velocity deficit near the floor, characteristic of a wake flow region where the recirculation bubble has velocity reversal near the wall (see $O_4$). The spanwise ($y^{*}$) velocity profiles in Fig. 9[b(iv)] further illustrate the lateral profiles of the velocity at a wall-normal height of $z^{*} = 0.5$. These profiles show a symmetric velocity distribution about the spanwise centerline ($y^{*} = 0$), with flow deficit near the centerline showing the low-momentum wake region and then the velocity increasing outwards.

**Fig. 11:**
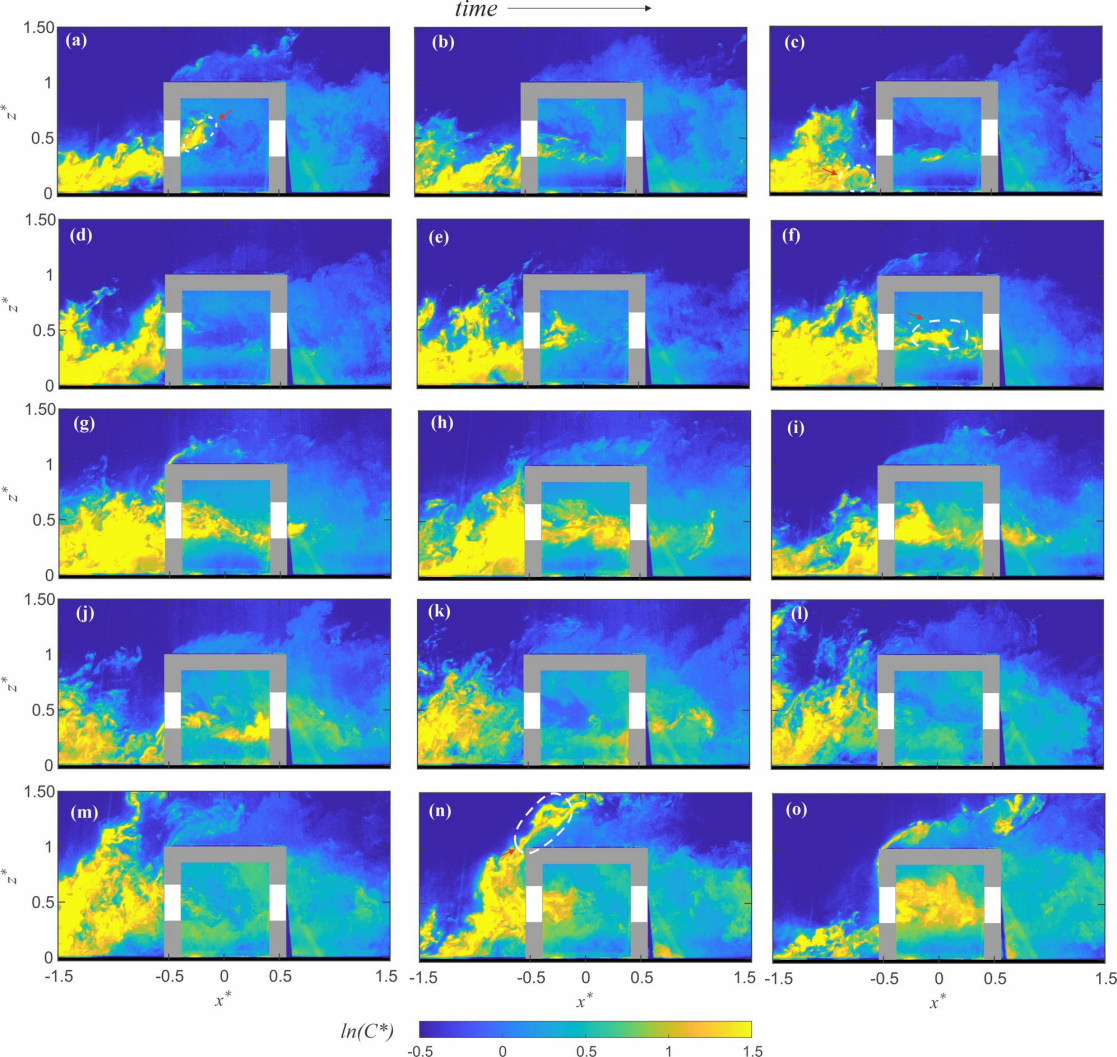
Instantaneous non-dimensional scalar (dye) concentration ($C^{*}=CAU_{{{\text{Ref}}}}/Q_S$) fields are shown from the water tunnel measurements at a time interval of 0.2 s. The flow is from left to right. The scalar concentration is presented on a natural logarithmic scale. The color bar, using a blue-to-yellow gradient, indicates increasing dye concentration

Following the analysis of the mean streamwise velocity, Fig. 8b presents the distribution of in-plane turbulent kinetic energy (TKE) $[{(\overline{u^{\prime }u^{\prime }}+\overline{w^{\prime }w^{\prime }})/U^2_{\text{Ref}} })]$ within the spanwise central $x-z$ plane. This figure reveals regions characterized by significant turbulence generation and mixing, particularly in proximity to and within the hollow cube. Within the cube, both the interfaces of the recirculation regions and the central jet exhibit elevated TKE levels. This phenomenon is further illustrated by wall-normal profiles in Fig. 10[a(ii),b(ii)], which depict the wall-normal variations in the turbulence intensity at different streamwise locations $I_1 - I_5$. The profiles show a significant increase in turbulent fluctuations indoors compared to the upstream outdoor locations. This is expected due to the large velocity fluctuations in the indoor space, which would have implications for the mixedness of the scalar within the indoor environment. Outside the cube, it is evident that the shear layer above the cube (for $z^{*} > 1$) is a critical region for turbulence production, as indicated by the larger TKE in Fig. 8b. This region plays a dominant role in the overall energy transfer from the mean flow to the recirculation region above the rooftop. The shear layer could also be important for scalar exchange, as the interaction between the fast-moving free-stream flow and the slower recirculating flow creates mixing effects. However, in the present scenario, the scalar passing over the cube would be substantially smaller, and hence, the role of this rooftop region on outdoor scalar transport would not be important, as will be discussed later. Downstream of the cube, both the 2-D map in Fig. 8b and profiles in Fig. 10[a(iii),b(iii)] clearly indicate that turbulence intensity is higher compared to the upstream conditions. This increase is due to vortex shedding and large-scale turbulent mixing occurring in the wake.

The strong agreement in the wind tunnel (LDA) and the water tunnel measurements (PIV) in capturing flow characteristics for both indoor and outdoor environments clearly confirms the consistency across the experimental setups and data acquisition methods.

### Instantaneous concentration field

We now characterize the scalar concentration field in both indoor and outdoor environments, providing insights into its spatial distribution and temporal variations. These insights are crucial for understanding scalar transport and mixing in a typical cross-ventilated flow system with an outdoor pollutant source.

We begin with Fig. 11 showing fifteen instantaneous snapshots (labeled ‘(a to o)’) of the instantaneous dye concentration ($C^{*}=CAU_{{{\text{Ref}}}}/Q_S$) in the $x-z$ plane at the spanwise center $y^{*} = 0$, from the water tunnel. Each of the scalar fields presented was recorded at time intervals of 0.2 seconds. In Fig. 11a, the scalar (dye)-laden flow is approaching the cube, where the high scalar concentration is evident from the yellow regions upstream to the cube. A fraction of this incoming scalar parcel is entrained into the cube through the upstream opening. This entrained scalar is seen inside the cube near the upstream opening, marked with a dashed white contour. Outside the cube, some portion of the oncoming scalar gets trapped in the recirculation zone upstream of the cube. This is apparent in Fig. 11c, where the scalar parcel inside the recirculating region is highlighted by a white dashed contour. The presence of this recirculation zone could play a role in scalar transport dynamics, potentially affecting both the scalar bypassing the cube and the amount that enters the indoor space. As time progresses, more scalar parcels approach the cube, e.g., as shown in Fig. 11d–g. It can be noted that most of the incoming scalar bypasses the cube, flowing around it, while a relatively smaller portion enters through the upstream opening. Figure 11f highlights a scalar parcel inside the cube, marked with a dashed white contour, which is primarily concentrated in the middle of the cube. Inside the cube, the scalar parcel is primarily advected by the internal central jet-like flow that passes along the midsection of the cube, through the openings. This process is evident in Fig. 11g,h, where the scalar is convected downstream within the cube. Simultaneously, since the jet interacts with the upper and lower recirculation regions, the scalar within this jet could also undergo exchange across these regions. In this process, some scalar is entrained from the jet into these recirculation regions, while scalar from these zones (trapped from earlier times) is also transported back into the jet. This continuous exchange contributes to the mixing of the scalar inside the cube. It may be noted that outdoors, while most of the oncoming scalar is transported around and through the cube, occasionally scalar parcels travel over the top of the cube, as shown in Fig. 11m,n,o, where the scalar parcel moving over the cube is marked with a dashed contour. Such events, however, are relatively less frequent. Downstream of the cube, the scalar that exits through the downstream opening, contributes to the wake region along with the scalar that bypasses the cube. The concentration in the wake is significantly weaker due to diffusion primarily from the larger velocity fluctuations, seen previously in Figs. 8b & 10[a(iii), b(iii)].

These broad observations provide insights into the scalar exchange in a cross-ventilated configuration. In the subsequent sections of this manuscript, we will further analyze the mean (time-averaged) and time-varying characteristics of the scalar within and around the cube. Additionally, we will examine the advective and turbulent scalar transport that drives scalar exchange processes, providing a more comprehensive understanding of pollutant dispersion.

### Concentration mean and variance

**Fig. 12:**
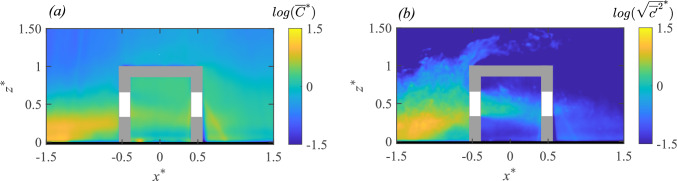
Time-averaged: **a** dye concentration (${\overline{C}}^{*}=\overline{C}AU_{{{\text{Ref}}}}/Q_S$, in natural logarithmic scale), and** b** concentration variance (${\sqrt{\overline{{c^{\prime }}^2}}}^{*}=\sqrt{\overline{{c^{\prime }}^2}}AU_{{{\text{Ref}}}}/Q_S$, in natural logarithmic scale), are shown in the center plane from water tunnel measurements. The area average of the indoor ‘time-averaged concentration’ (${\overline{C_A}}^{*}$) is given in Table 3 (Appendix)

**Fig. 13:**
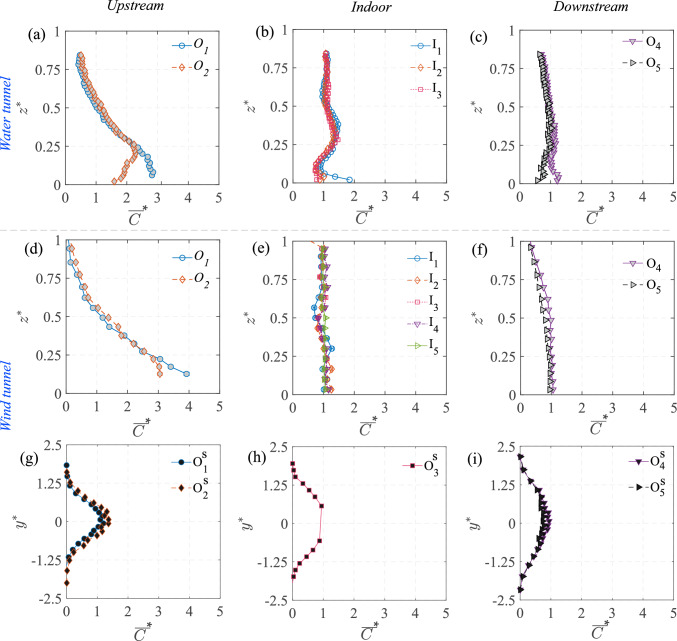
The wall-normal ($z^{*}$) profiles of time-averaged concentration (${\overline{C}}^{*}=\overline{C}AU_{{{\text{Ref}}}}/Q_S$), measured upstream to the model (**a**,**d**), inside (**b**,**e**), and downstream (**c**,**f**), are shown from the water tunnel and wind tunnel measurements. Also shown in ‘(**g**,**h**,**i**)’ are the spanwise ($y^{*}$) concentration profiles at different streamwise locations, from the wind tunnel

The 2-D map (in $x-z$ plane) of the time average of the instantaneous scalar concentration (${\overline{C}}^{*}={\overline{C}}AU_{{{\text{Ref}}}}/Q_S$) from the water tunnel is presented in Fig. 12a. In addition, the wall-normal profiles of the concentration at different locations from the water tunnel (using PIV) and the wind tunnel (FFID) are shown in Fig. 13. Similar to the velocity profiles discussed earlier, these are shown at multiple locations: upstream of the cube at $x^{*} = -1.5$ and $x^{*} = -1$ (denoted as $O_1$ and $O_2$) in Fig. 13a,d; inside the cube along five vertical lines ($I_1, I_2, I_3, I_4, I_5$) in Fig. 13(b,e); and downstream of the cube at $x^{*} = 1$ and $x^{*} = 1.5$ (denoted as $O_4$ and $O_5$) in Fig. 13(c,f). Additionally, spanwise (along $y^{*}$) concentration profiles were taken (Fig. 13(g,h,i)) in the wind tunnel at a fixed wall-normal height of $z^{*} = 0.5$, measured upstream ($O^S_1, O^S_2$), on either side of the cube ($O^S_3$) and downstream ($O^S_4, O^S_5$). While the mean concentration field provides insight into the overall scalar distribution, the variance highlights the dynamic nature of turbulence-driven concentration fluctuations.

In Fig. 12a, the mean (time-averaged) concentration (${\overline{C}}^{*}$) distribution exhibits distinct characteristics in different regions. Upstream of the cube ($x^{*} < -0.5$), the concentration is mostly localized near the ground and then gradually increases vertically as the scalar-laden flow approaches the cube, as seen in both the 2-D map (in Fig. 12a) and the vertical profiles [at $O_1$ and $O_2$, in Fig. 13a,d]. Inside the cube, ${\overline{C}}^{*}$ is a bit lower than the upstream concentration since only a portion of the oncoming scalar parcels are seen entering (intermittently) the cube. Within the cube, the concentration is seen to be nearly uniform (well-mixed), which would be due to the large turbulent intensity, thus breaking the scalar parcels and enhancing mixing. Far downstream of the cube ($x^{*} \gg 0.5$), the concentration decreases as the scalar disperses into the wake region, which is also evident from the wall-normal profiles (at $O_5$) in Fig. 13c,f).

The spanwise concentration profiles in Fig. 13g,h,i from the wind tunnel provides further insight into the lateral scalar dispersion. Upstream of the cube at $O^S_1 \quad \& \quad O^S_2$ (in Fig. 13g), the concentration follows a nearly symmetric Gaussian distribution. Downstream ($O^S_4, O^S_5$, 13i), the pollutant spread is much broader than upstream, consistent with the fact that the wake downstream enhances the lateral mixing and spread. Now, another aspect to be noted is the nearly similar wall-normal concentration inside the cube in Fig. 13b,e, from both the wind and water measurements. In addition, the concentration distribution and magnitudes are nearly similar at different streamwise positions, as seen from the profiles ($I_1$ to $I_3$) in Fig. 13. Lastly, these values in the center plane are closer to the wall-normal profiles taken at spanwise offset positions ($I_4$ & $I_5$). These indicate a nearly well-mixed scalar distribution (volumetric) in the indoor environment. In addition to these, another notable characteristic would be the comparison of scalar spread outdoor with and without the cube, shown in Fig. 7, in terms of the standard deviations ($\sigma _{{\overline{C}}^{*},y^{*}}$, $\sigma _{{\overline{C}}^{*},z^{*}}$). These were obtained previously from the spanwise and wall-normal spread of the plume using Eqs. 3 and 4. As noted from Fig. 7, ‘$\sigma _{\overline{C},y^{*}}$’ increases by $\approx$20% at upstream ($x^{*}=-1$) compared to the no-cube case, while it increases by $\approx$100% downstream ($x^{*}=-1.5$).

**Fig. 14:**
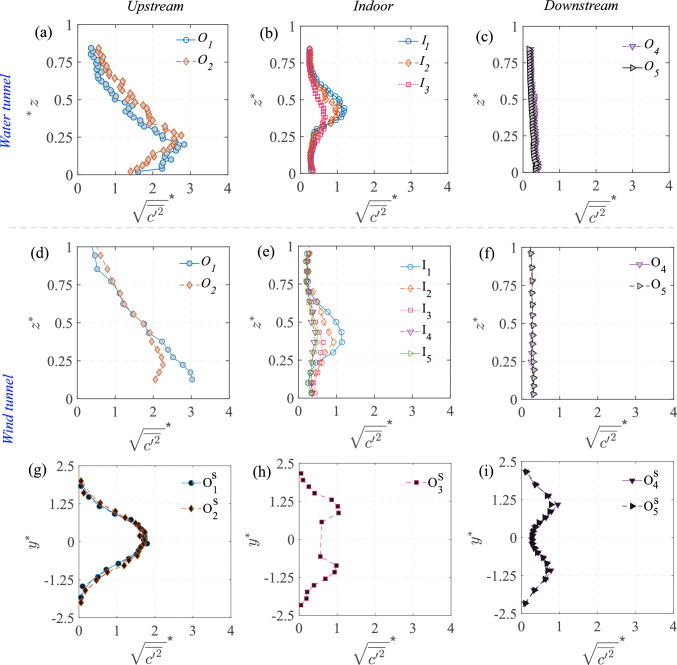
The wall-normal ($z^{*}$) profiles of the variance, ${\sqrt{\overline{{c^{\prime }}^2}}}^{*}=\sqrt{\overline{{c^{\prime }}^2}}AU_{{{\text{Ref}}}}/Q_S$, of the instantaneous concentrations ‘${{C}}^{*}={C}AU_{{{\text{Ref}}}}/Q_S$,’ measured upstream to the model (**a**,**d**), inside (**b**,**e**), and downstream (**c**,**f**), are shown from the water tunnel and wind tunnel measurements. Also shown in (**g**,**h**,**i**) are the spanwise ($y^{*}$) concentration variance profiles, at different streamwise locations, from the wind tunnel

The concentration variance map (${\sqrt{\overline{c^{\prime }c^{\prime }}}}^{*}=\sqrt{\overline{c^{\prime }c^{\prime }}}AU_{{{\text{Ref}}}}/Q_S$) is shown in Fig. 12b, along with the wall-normal and spanwise profiles in Fig. 14. These help us identify the regions where fluctuations in scalar concentration are most significant. Upstream of the cube, say at $O_2$ ($x^{*} \approx -1.5$) in Fig. 12b, the variance is relatively higher compared to both indoor and downstream regions, which is also corroborated by the profiles in Fig. 14a,d. Inside the cube, the variance remains low and relatively uniform (Fig. 14b,e), except for the central jet area. In the wake downstream, variance decreases even further (Fig. 14c,f). The spanwise distribution from the wind tunnel, presented in Fig. 14g–i, provides additional insights into the lateral dispersion. Upstream (at $O^S_1, O^S_2$), the variance exhibits a nearly Gaussian shape, whereas at downstream ($O^S_4, O^S_5$), the variance follows a bi-modal distribution.

When comparing the water tunnel and wind tunnel data for the wall-normal profiles, their substantial similarities in both the mean concentration and its variance clearly show that the broad mechanisms governing the dispersions are captured by both the measurement techniques, even though the Schmidt number is different. This indicates that, for this flow, turbulent mixing is more influential than molecular mixing. The results also highlight the strength of both the facilities and techniques in evaluating the dynamics of scalar dispersion in urban flows.

### Scalar time traces

**Fig. 15:**
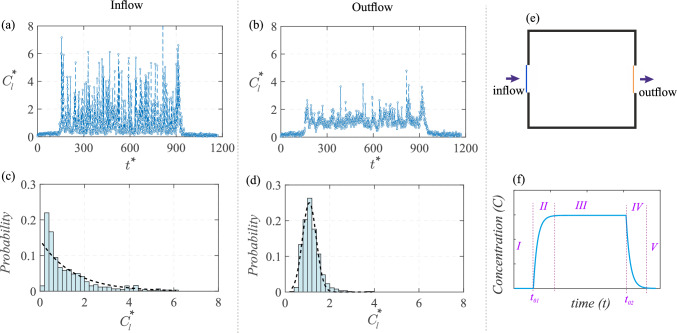
The instantaneous scalar concentration ($C^{*}_l={C_l}AU_{{{\text{Ref}}}}/Q_S$) is shown with time ($t^{*}=tU_{{{\text{Ref}}}}/H$) at the inlet window to the model (in ‘a’) and at the outlet window (in ‘b’). Here $C_l$ is the (dimensional) instantaneous scalar concentration averaged over a line, as demarcated in blue () and yellow () for the inflow and outflow, respectively, as in the schematic ‘e.’ Also shown are the corresponding probability distributions of $C^{*}_l$ in ‘c,d.’ These are from water tunnel measurements. **f** Schematic representing the typical profile of scalar concentration against time and the respective stages involved

We now focus on characterizing the scalar concentration time traces. It may be noted that all the measurements are performed after the flow and scalar field have reached a statistically steady-state condition. Before we proceed, it is first worth noting that the concentration time trace at any location can be seen to consist of five distinct stages (‘*I*’ to ‘*V*’), as illustrated in Fig. 15f. In the figure, the statistically steady-state concentration in the stage ‘*III*’ is reached following an initial no scalar injection stage ‘*I*,’ and a scalar build-up stage ‘*II*’ spanning from the beginning of scalar development at $t_{01}$ up to the time it takes to reach a statistically steady-state concentration. Moving further in time, stage ‘*III*’ ends as the scalar injection is turned off and then follows an exponential decay (from $t_{02}$) in concentration in stage ‘*IV*,’ and then, the onset of stage ‘*V*’ after the scalar is entirely flushed out. Among the five stages, our focus is on stage *III* which governs long-term exposure, and then stage *IV* which reflects the time scales associated with flushing out of indoor pollutants.

#### Scalar time traces at inflow/outflow windows

We now characterize the scalar at the upstream (inflow to the cube) and downstream (outflow) window openings. We begin with the temporal scalar concentration variations from the water tunnel measurements, as shown in Fig. 15a,b. To capture the concentration at the inflow and outflow windows, at each time instances, the measured concentration values are averaged along vertical lines (spanning from $z^{*}=0.325-0.675$) positioned immediately upfront to the inlet window ($x^{*}=-0.55$) and downstream of the exit window ($x^{*}=0.55$), as demonstrated by a schematic in Fig. 15e. At the inlet, this line-averaged instantaneous concentration (${C_l}^{*}=C_lAU_{{{\text{Ref}}}}/Q_S$) in Fig. 15a shows that ${C_l}^{*}$ exhibits large fluctuations with sharp peaks and troughs over time ($t^{*}=tU_{{{\text{Ref}}}}/H$). However, the peak ${C_l}^{*}$ events are less frequent due to the intermittent (inflow) bursts of pollutant influx. Such characteristics are also evident from the probability distribution of ${C_l}^{*}$ in Fig. 15c, where the histogram shows a right-skewed distribution with the majority of concentration values clustered around a lower range of ${C_l}^{*}\approx 1$, indicating that low pollutant concentration parcel influx to the window occurs more frequently. It may be noted that the histogram is computed over stage ‘*III*’ as the statistically steady-state concentration is attained.

Compared to the inlet, the fluctuations in $C^{*}_l$ at the outlet are relatively smaller (in Fig. 15b), suggesting that the scalar passing through the cube had already undergone a mixing process inside the cube. This behavior is again reflected in the histogram in Fig. 15d, showing a nearly Gaussian distribution with a slight skew to the right. The clustering of $C^{*}_l$, however, is closer, indicating a more stabilized concentration range over time. These suggest that the scalar dispersion process increases the homogenization of the scalar as the flow passes through the cube. This would be attributed to a number of reasons, for example, larger fluctuations in the velocity inside the cube (i.e., higher $\overline{{u^{\prime }}^2}$, seen Fig. 10b) help to better mix the concentration by breaking up the large concentration scalar parcels. It may be noted that the mean (time average) of ${{C}}^{*}_l$ at both the inflow and outflow are nearly the same (${\overline{C}}^{*}_l\approx 1.2$), as also indicated in Table 3 (Appendix), with some minor difference, possibly due to some variations in the scalar concentration in the out-of-plane direction. We will discuss these aspects related to scalar concentration, mixing and out-of-plane inhomogeneity in the following sections.

**Fig. 16:**
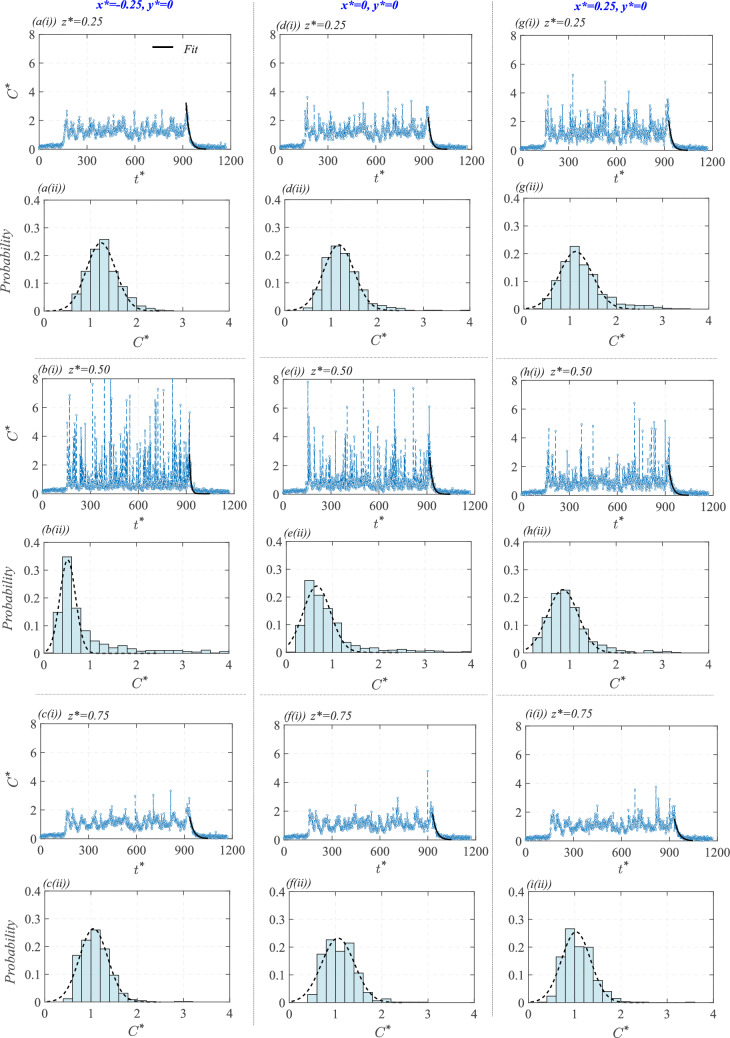
Time traces and probability distributions of the instantaneous concentration ($C^{*}$) are shown at nine different points within the cube, comprising three vertical heights ($z^{*} = 0.25, 0.5, 0.75$) across three streamwise positions of: (**a**,**b**,**c**) $x^{*}=-0.25$, (**d**,**e**,**f**) $x^{*}=0$, and (**g**,**h**,**i**) $x^{*}=0.25$; these all are taken at $y^{*}=0$. These measurements are from the water tunnel facility. Also shown is an exponential fit in the time traces for concentration decay (black solid line, ). From the fit, the corresponding decay constant values have been obtained and are shown in Fig. 19

**Fig. 17:**
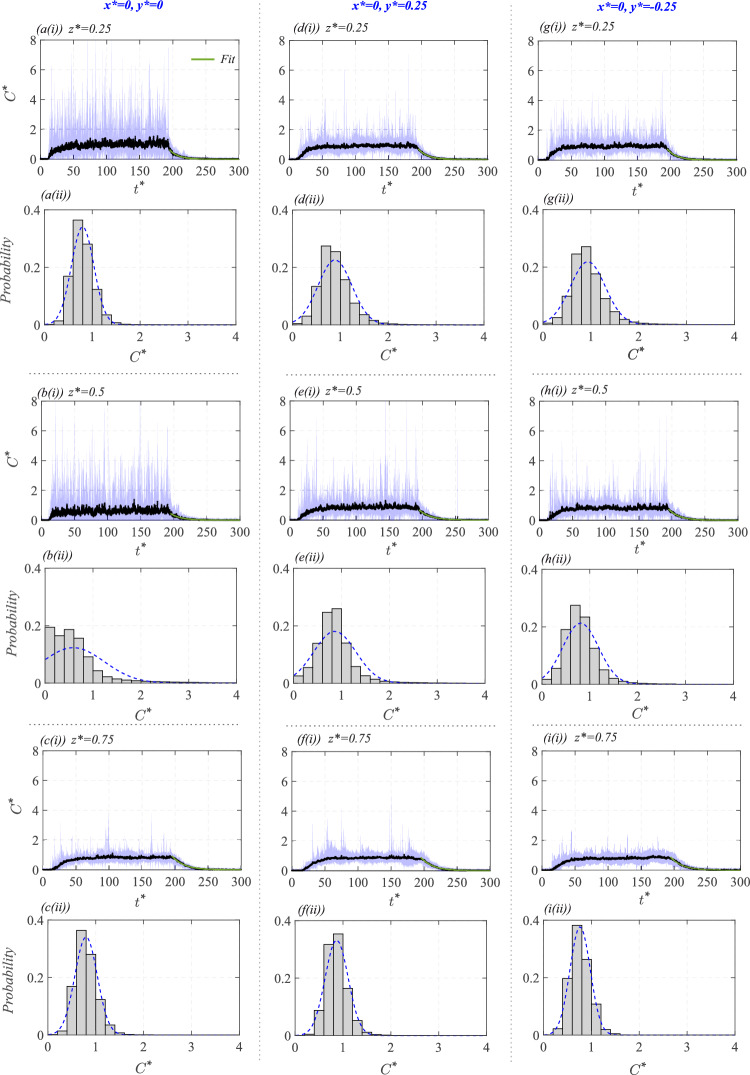
Time traces and probability distributions of the instantaneous concentration ($C^{*}$) are shown at nine different points within the cube, comprising three vertical heights ($z^{*} = 0.25, 0.5, 0.75$) across three spanwise positions of: (**a**,**b**,**c**) $y^{*}=0$, (**d**,**e**,**f**) $y^{*}=0.25$, and (**g**,**h**,**i**) $y^{*}=-0.25$; these all are taken at $x^{*}=0$. These measurements are from the wind tunnel facility. Also shown in the time traces is an exponential fit (green solid line,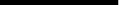) to the concentration decay data. From the fit, the corresponding decay constant values have been obtained and are shown in Fig. 19

#### Scalar time traces at different indoor locations

To further understand the time-varying nature of indoor concentration, we present time traces and probability distribution of the instantaneous concentration ($C^{*}$) at nine different points indoors from the water tunnel measurements, as given in Fig. 16. These measurement locations comprise three vertical heights ($z^{*} = 0.25, 0.5, 0.75$) across three streamwise positions ($x^{*} = -0.25, 0, 0.25$), all taken at $y^{*}=0$. These positions encompass both the lower and upper recirculation regions ($R_{low}$ and $R_{up}$), as well as the central (jet-like) area of the cube’s interior. At the lowest vertical point of $z^{*} = 0.25$, which corresponds to the near-ground recirculation region ($R_{low}$), the time traces in Fig. 16[a(i),d(i),g(i)] show some intermittent bursts of concentration (in stage ‘*III*’) at all three streamwise locations ($x^{*} = -0.25, 0, 0.25$).

The concentration fluctuation in the time traces in Fig. 16[a(i),d(i),g(i)] indicates that the scalar is entrained into the $R_{low}$ in an unsteady manner, with periods of low concentration followed by an increase when parcels of the scalar are transported into the region and vice versa. The probability distributions in Fig. 16[a(ii),d(ii),g(ii)] again confirm these observations, showing slightly skewed (to the right) distributions, implying a strong likelihood of low concentration values, however, with occasional larger values. Comparing across the three streamwise locations ($x^{*}=-0.25, 0, 0.25$), the fluctuations are most pronounced downstream at $x^{*} = 0.25$ (in 16[g(i)]), also seen from the relatively more skewed nature of the histogram in Fig. 16[g(ii)]. Presently, for computing the histograms, the values of $C^{*}$ corresponding to the statistically equilibrium concentration region (stage *III*) have been considered. It is worth noting that, in addition to scalar exchange events occurring across the recirculation zones (both upper and lower) and the central region, there will also be out-of-plane scalar transport, which also contributes to time-varying scalar fluctuations.

In the vertical middle region ($z^{*} = 0.5$), which corresponds to the centerline in the core area of the oscillating jet, the time traces in Fig. 16[b(i),e(i),h(i)] exhibit much stronger fluctuations compared to those in $R_{low}$ ($z^{*} = 0.25$) that have already been discussed. For a better comparison, the time-averaged concentration ($\overline{C}^{*}$) and the standard deviation of the instantaneous concentration ($\sigma _{C^{*}}$) are shown in Table 3 for the different measurement points. The large fluctuations in the central region can be attributed, firstly, to the presence of intermittent large concentrated scalar parcels within the jet region, and secondly, to the significant fluctuations in velocity found in this area. The most considerable concentration fluctuation occurs at $x = -0.25$, which is closer to the inflow window (16e(i)). At $z^{*} = 0.5$, the histograms in Fig. 16[b(ii),e(ii),h(ii)] show broader spreads compared to those seen in the lower region at $z^{*} = 0.25$. The most skewed distributions are observed near the inlet window at $x^{*} = -0.5$. Moving vertically, at $z^{*} = 0.75$ in $R_{up}$, the time traces in Fig. 16[c(i),f(i),i(i)] show reduced fluctuations compared to the middle region and resemble those at $z^{*} = 0.25$. It is, however, worth noting that the concentration fluctuations in $R_{up}$ are slightly lower than those in $R_{low}$. This observation is again supported by the probability distributions in Fig. 16[c(ii),f(ii),i(ii)], depicting relatively symmetric distributions compared to $R_{low}$.

In summary, the contrast between the jet-dominated central region ($z^{*}=0.5$) and the recirculation-dominated lower and upper regions ($z^{*} = 0.25, 0.75$) reveals that the central areas exhibit the strongest concentration fluctuations. These fluctuations arise from periodic jet oscillations and the presence of intermittent, largely concentrated scalar parcels, which lead to a broader and skewed probability distribution. In contrast, the upper and lower recirculation regions experience relatively lower velocity fluctuations and less frequent occurrences of large concentration scalar intrusions from the jet region. As a result, these regions have more symmetric concentration distributions. Furthermore, the comparison across different streamwise locations shows that the upstream region ($x^{*}=-0.25$) experiences more frequent bursts of high concentration. In contrast, the downstream region (e.g., $x^{*}=0, 0.25$) follows more sustained but lower-amplitude fluctuations. Overall, these findings indicate that scalar transport within the cube is heavily influenced by events such as the complex interplay between recirculation regions and oscillating jets.

The time traces discussed thus far correspond to measurements conducted in the water tunnel. We now proceed with Fig. 17 presenting measurements from the wind tunnel facility. These measurements are at three wall-normal locations of $z^{*} = 0.25$, 0.5, and 0.75, measured at different spanwise locations of $y^{*} = 0$, 0.25, and $-0.25$, all taken along the streamwise center ($x^{*} = 0$). We now compare the time traces from the wind tunnel [Fig. 17a–c] and water tunnel [Fig. 16d–f] measurements taken at $(x^{*}, y^{*})=(0,0)$. Before beginning the comparison, it is worth noting that in the water tunnel, a single long-duration measurement (for stage ‘*III*’) was performed, comprising approximately 1500 instantaneous samples and spanning a window of $t^{*} \approx 150$–900. In contrast, in the wind tunnel, more than ten shorter runs were conducted, each covering $t^{*} \approx 10$–180. In Fig. 17, the average over ten wind tunnel runs is presented (black line), accompanied by a shaded band indicating variability across runs (based on $>30,000$ instantaneous samples). At $z^{*} = 0.25$, the concentration fluctuations in the wind tunnel (in Fig. 17a(i)) appear to be marginally higher than those in the water tunnel (in Fig. 16d(i)). However, the corresponding histograms in Fig. 16d(ii) &  17a(ii) reveal that the concentration variance in the wind tunnel is narrower. Furthermore, the most probable value in the wind tunnel occurs around $C^{*} \approx 0.75$, whereas in the water tunnel, it is around $C^{*} \approx 1.1$. These trends are consistent at other wall-normal locations of $z^{*} = 0.5$ and 0.75 (Figs. 16(e,f) and 17(b,c)), with the wind tunnel exhibiting a comparatively narrower concentration variance and the mean concentration $\overline{C}^{*}$ being slightly lower than in the water tunnel. These relatively minor differences between the two facilities may be attributed to several factors, for example, the much extensive dataset in the wind tunnel, as well as the lower Schmidt number ($Sc \approx 1$), implying more mixing due to faster molecular diffusion. In contrast, the higher Schmidt number in the water tunnel ($Sc \approx 2500\pm 300$) leads to slower molecular diffusion and thus would result in comparatively lowered mixing and, consequently, a broader concentration bandwidth. Despite these subtle differences, it is important to note that the overall trends and magnitudes of concentration, including its temporal evolution and spatial distribution, are in close agreement across both facilities.

**Fig. 18:**
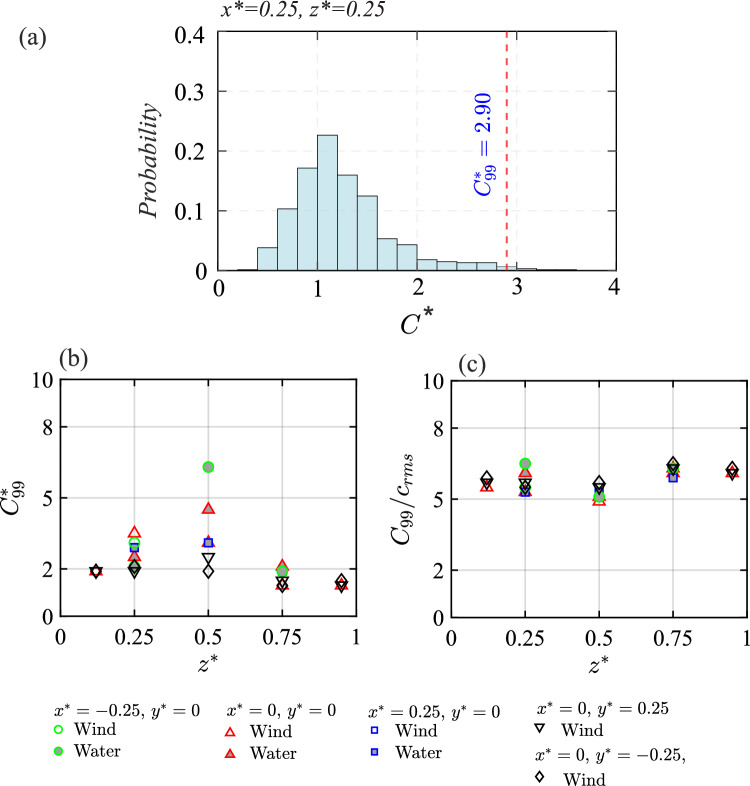
**a** The probability distribution of the instantaneous concentration ($C^{*}$), with $C^{*}_{99}$ demarcated, is shown at $x^{*}, z^{*}=0.25, 0.25$ (in center plane).** b** The non-dimensional peak concentration ($C^{*}_{99}$), and** c** peak concentration normalized by the concentration fluctuation root-mean-square ($C_{99}/c_{rms}$), are plotted with the corresponding vertical location ($z^{*}$), taken at different streamwise/spanwise ($x^{*}$, $y^{*}$) positions

#### Peak concentration

To gain understanding of high concentration events indoors, we now discuss the peak concentration levels, measured at different indoor locations as in Figs. 16 and 17. To define peak concentration, a new parameter, $C^{*}_{99}=C_{99}AU_{{{\text{Ref}}}}/Q_{S}$, is introduced which is defined as the concentration value that is exceeded 1% of the time (in stage *III*). For illustration, $C^{*}_{99}$ is marked in Fig. 18a showing the probability distribution of $C^{*}$ at $(x^{*}, z^{*})=0.25, 0.25$ in the spanwise center plane. In Fig. 18b, $C^{*}_{99}$ is further plotted with $z^{*}$, for different streamwise/spanwise ($x^{*}$, $y^{*}$) positions. The figure shows larger peak concentrations around $z^{*}\approx 0.5$, and this is due to the presence of largely concentrated scalar parcels in the mid-height regions. The upper and lower recirculation regions exhibit relatively lower $C^{*}_{99}$, with the lower regions being slightly more susceptible to peak concentration exposure than the upper one. To understand the peak concentration in connection with the concentration fluctuations, $C_{99}$ is normalized by the root-mean-square of the concentration variance ($c_{rms}$). As can be seen in Fig. 18c, $C_{99}/c_{rms}$ nearly collapses to be broadly around $\approx 5-6$, in line with the previous studies on indoor pollutant dispersions (e.g., Lim et al. (2024)).

#### Scalar mixedness

It may be noted that the time-resolved measurements discussed thus far have been limited to the streamwise $x-z$ plane situated at spanwise center ($y^{*} = 0$). To obtain a more comprehensive understanding of the concentration within the indoor volume, additional measurements were carried out at spanwise offsets of $y^{*} = 0.25$ and $-0.25$ (denoted as $I_4$ and $I_5$, see Fig. 5), as given in Fig. 17(d-i). These figures present a comparison between the two spanwise locations at various wall-normal positions of $z^{*} = 0.25, 0.5, 0.75$. The results indicate nearly identical behavior in both the time traces and their corresponding histograms across the two spanwise offset positions, showing symmetry about the center plane. Now, looking into the variations in the time traces of $C^{*}$ with $z^{*}$, it is noticeable that concentration fluctuations increase with $z^{*}$, reaching a higher value at mid-height. This trend is also reflected in the standard deviation of instantaneous concentration values ($\sigma _{C^{*}}$), as was reported in Table 3. Notably, this vertical trend is consistent with the wall-normal variations of time-varying $C^{*}$ observed in the center plane ($y^{*} = 0$) in Figs. 16  & 17a–c. Furthermore, the time-averaged concentration ($\overline{C}^{*}$) at $y^{*} = 0.25$ is found to be nearly identical to those measured at the center plane, which was previously noted in Fig. 13e (also given in Table 3). Together, these observations suggest that the scalar is nearly well-mixed spanwise (within the indoor volume). It is worth noting that this is unlike the case of an indoor scalar source reported recently by Biswas and Vanderwel (2024), where significant out-of-plane variations, by a factor up to 20, in the scalar concentration were noted.

**Fig. 19:**
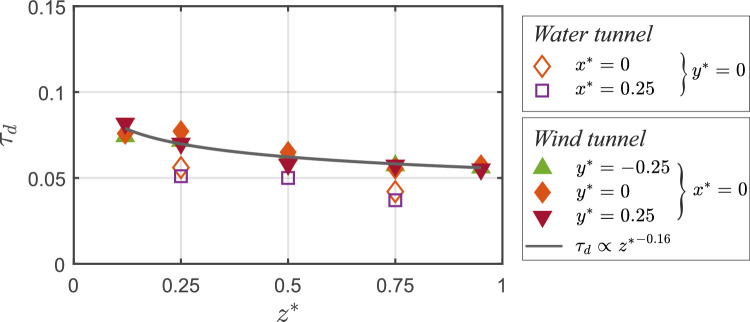
The decay constant ($\tau _d$, from Equation 5) is plotted with the corresponding vertical location ($z^{*}$), taken at different streamwise/spanwise ($x^{*}$, $y^{*}$) positions

#### Decay time scale

We now focus on another key parameter foWe now try understanding the scalar transport mechanisms based on tr assessing ventilation effectiveness, the concentration decay time scale. This metric is particularly important in indoor ventilation studies, as it quantifies the rate at which contaminants are removed. In all time traces presented so far, it is observed that following the statistically steady-state concentration in stage *III*, the concentration begins to decay (exponentially) in stage *IV*. In this decay stage, we now quantify the rate of reduction in concentration across all time traces shown in Figs. 16  & 17, following an exponential fit:

5 $$\begin{aligned} C^{*}(t) = \overline{C}^{*} e^{-\tau _d t^{*}} \end{aligned}$$

Here $\overline{C}^{*}$ is the time-averaged concentration over stage ‘*III*,’ and $\tau _d$ represents the decay constant, obtained via least-squares fitting of the data in stage ‘*IV*’ using Eq. 5. The resulting exponential fits are shown in Figs. 16 and 17, using a solid black line (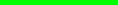) for the water tunnel and a solid green line (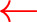) for wind tunnel. The corresponding ‘$\tau _d$’ values are plotted in Fig. 19 and also summarized in Table 4. As seen in the figure, $\tau _d$ from wind tunnel measurements at different spanwise positions ($y^{*} = -0.25$, 0, and 0.25) exhibit a close collapse, suggesting minimal spanwise variation for the decay rate. Furthermore, $\tau _d$ is observed to increase with going downwards, as $\tau _d \propto {z^{*}}^{-0.16}$, indicating a faster decay rate near the floor and slower around the ceiling region thus a longer exposure risk to contaminants. Also notable is that the decay constants obtained from the water tunnel data closely follow those from the wind tunnel, with only minor discrepancies. These small differences are likely attributable to the fact that in the wind tunnel, Eq. 5 was applied to the ensemble-averaged $C^{*}$ (from ten runs), whereas in the water tunnel, the fit was performed on a single run. Taken together, the results from both experimental facilities consistently indicate that contaminant removal is faster near the floor and slower toward the ceiling. These insights would have implications for the design and optimization of indoor ventilation systems, offering guidance for improving air quality.

### Advective and turbulent flux

**Fig. 20:**
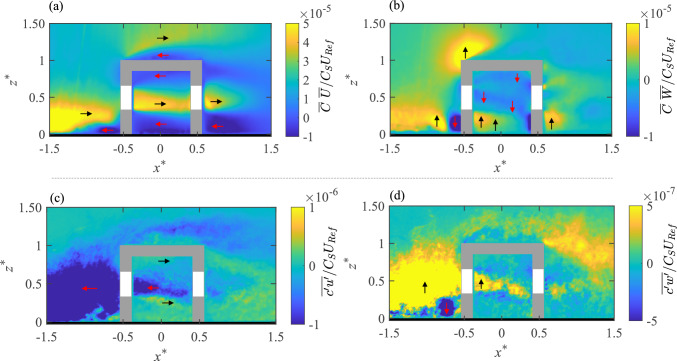
**a** Streamwise advective flux ($\overline{C}{\hspace{0.05cm}}\overline{U}/C_SU_{{{\text{Ref}}}}$), **b** wall-normal advective flux ($\overline{C}{\hspace{0.05cm}}\overline{W}/C_SU_{{{\text{Ref}}}}$),** c** streamwise turbulent flux ($\overline{c^{\prime }u^{\prime }}/C_SU_{{{\text{Ref}}}}$), and** d** wall-normal turbulent flux ($\overline{c^{\prime }w^{\prime }}/C_SU_{{{\text{Ref}}}}$), shown from water tunnel measurements. These measurements are all performed in the streamwise $x-z$ plane along the spanwise center position $y^{*}=0$

**Fig. 21:**
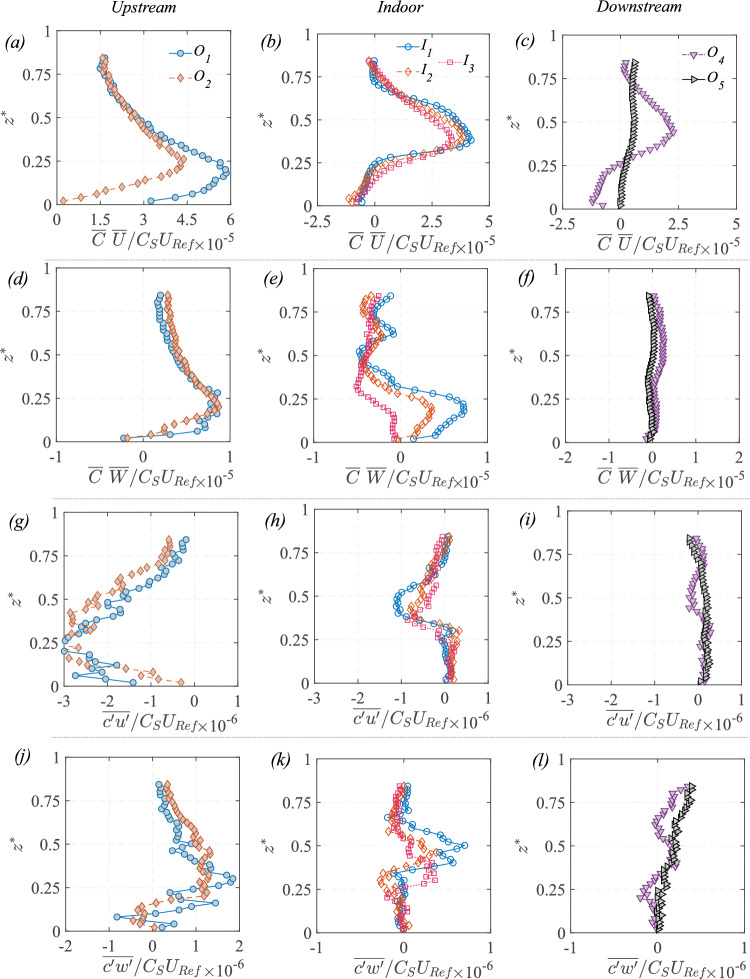
Wall-normal ($z^{*}$) profiles of (**a**–**c**) streamwise advective flux ($\overline{C}{\hspace{0.05cm}}\overline{U}/C_SU_{{{\text{Ref}}}}$), (**d**–**f**) wall-normal advective flux ($\overline{C}{\hspace{0.05cm}}\overline{W}/C_SU_{{{\text{Ref}}}}$), (**g**–**i**) streamwise turbulent flux ($\overline{c^{\prime }u^{\prime }}/C_SU_{{{\text{Ref}}}}$), and (**j**–**l**) wall-normal turbulent flux ($\overline{c^{\prime }w^{\prime }}/C_SU_{{{\text{Ref}}}}$), shown at the upstream, indoor, and downstream to the model, from water tunnel measurements. These profiles are extracted from Fig. 20

To gain deeper insight into scalar distribution, we now examine scalar transport in terms of two key components: the advective flux, which represents the bulk transport of scalar by the mean flow, and the turbulent flux, which accounts for transport due to velocity and concentration fluctuations. Figure 20 presents the spatial distribution of both advective and turbulent scalar fluxes in the $x-z$ plane along the spanwise center ($y^{*} = 0$), from water tunnel measurements. In addition, Fig. 21 shows wall-normal profiles of scalar fluxes at various streamwise positions: upstream of the cube ($O_1$, $O_2$), inside the cube ($I_1$, $I_2$, $I_3$), and downstream ($O_4$, $O_5$), all extracted from Fig. 20. The advective fluxes in Fig. 20a,b quantify the transport of scalar by the mean velocity field and are defined as $\overline{C} \, \overline{U}/(C_S U_{\textrm{Ref}})$ and $\overline{C} \, \overline{W}/(C_S U_{\textrm{Ref}})$, for the streamwise and wall-normal directions, respectively; here $\overline{U}$ and $\overline{W}$ are the time-averaged streamwise and wall-normal velocity, $\overline{C}$ is the time-averaged scalar concentration, and $C_S$ is the source concentration. In contrast with these, the turbulent fluxes in Fig. 20(c,d) represent transport due to the variance in velocity and concentration. These are computed as the time averages of the product of fluctuations, i.e., $\overline{c' u'}$ and $\overline{c' w'}$, where $c' = C - \overline{C}$, and $u'$ and $w'$ are the velocity fluctuations in the streamwise and wall-normal directions, respectively. To help better interpretation, directional arrows are used in Fig. 20: transport in the ‘$+x$’ direction is indicated by a black arrow ($\rightarrow$) and in the ‘$-x$’ direction by a red arrow (); similarly, transport in the ‘$+z$’ and ‘$-z$’ directions are represented by ($\uparrow$) and (), respectively.

We now try understanding the scalar transport mechanisms based on the flux maps in Fig. 20. In the region upstream of the cube, Fig. 20a shows that the scalar is primarily transported by a $(+)ve \,\,\overline{C} \, \overline{U}/C_S U_{{Ref}}$, showing the bulk movement of scalar toward the cube. Simultaneously, a relatively weaker $(+)ve \,\,\overline{C} \, \overline{W}/C_S U_{{Ref}}$ is evident in Fig. 20b, suggesting transport in the ‘$+\,z$’ direction. Conversely, within the upstream recirculation regions, $(-)ve \,\,\overline{C} \, \overline{U}/C_S U_{{Ref}}$ indicate reverse transport (in ‘$-x$’). These trends are also clearly reflected in the wall-normal profiles presented in Fig. 21a–f. Further insight is gained from the turbulent flux components shown in Fig. 20c,d, and their corresponding profiles in Fig. 21g,j. A notable $(-)ve\,\, \overline{c'u'}/C_S U_{{Ref}}$ and $(+)ve\,\,\overline{c'w'}/C_S U_{{Ref}}$ indicate local mixing of the incoming scalar due to velocity and concentration fluctuations.

Inside the cube, a strong $(+)ve\, \overline{C} \,\, \overline{U}/C_S U_{{Ref}}$ is observed in the mid-height region (Fig. 20a), illustrating dominant forward transport of scalar within the central jet-like flow. Within this region, $(-)ve\,\,\overline{c'u'}/C_S U_{{Ref}}$ indicate mixing, contributing to the scalar homogenization inside the jet. As scalar parcels are advected through the core of the cube, portions of them are likely entrained into the recirculation regions via vertical transport, as was discussed earlier in Fig. 11. This exchange is facilitated by $\overline{C} \, \overline{W}/C_S U_{{Ref}}$, which plays a critical role in mediating scalar transfer between the jet and both the recirculation regions. The vertical exchange is further enhanced by $(+)ve\,\,\overline{c'w'}/C_S U_{{Ref}}$, particularly evident in Figs. 20d and 21k, which indicates upward turbulent transport from the central jet into the upper recirculation region. Collectively, these observations highlight the coupled roles of advective and turbulent processes in scalar transport and distribution within and around the cube.

### Outdoor versus indoor source: a comparison

**Fig. 22:**
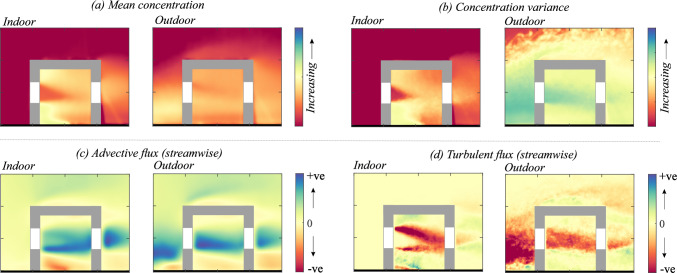
A qualitative comparison between indoor and outdoor source cases, for time-averaged:** a** concentration,** b** concentration variance,** c** streamwise advective flux, and** d** streamwise turbulent flux

We now present a direct comparison between the indoor and outdoor source configurations, revealing similarities and differences in the scalar dispersion and transport mechanisms. In the indoor source case reported by Biswas and Vanderwel (2024), the scalar is steadily released into the indoor environment. The released scalar is initially entrained within the lower recirculation region before being transported into the central jet. As the jet advects the scalar downstream, exchange of scalar occurs with the upper and lower recirculation regions, accompanied by re-entrainment from the recirculation zones back into the jet. During these events, pollutant removal is primarily governed by streamwise advection in the central jet, while turbulent and wall-normal advective fluxes facilitate scalar exchange between the recirculation zones and the jet. In the present outdoor source case, the scalar is transported toward the cube within the approaching turbulent boundary layer, dominated by streamwise advection, and enters the cube through the upstream opening where the scalar inflow is seen to be intermittent. A substantial fraction of the incoming scalar bypasses the interior entirely due to flow separation around the windward façade. Once inside, the scalar follows redistribution pathways similar to those in the indoor source case, with scalar exchange between the jet and the upper and lower recirculation zones promoted by turbulent and wall-normal advective fluxes.

The spatial concentration fields exhibit substantial differences between the two cases. For the indoor source, the indoor scalar is seen to occur primarily within the recirculation zones (Fig. 22) and is strongly inhomogeneous both streamwise and spanwise. In contrast, for the outdoor source, the indoor scalar concentration is nearly homogeneous, indicating better mixing as compared to the indoor case. The concentration variance is seen to be more pronounced in the central jet region due to intermittent large scalar parcel inflow events. Following source turning off, the indoor concentration decay rates are nearly identical for both cases ($\propto e^{-0.04t^{*}}$), indicating similar ventilation-driven pollutant flushing.

A comparison of the scalar flux terms shows that the streamwise and wall-normal advective fluxes show similar patterns in both cases, dominated by transport within the central jet. However, the turbulent counterparts are seen to be different. For the indoor source, the strongest streamwise and wall-normal turbulent fluxes occur at the interfaces between the jet and recirculation zones, whereas for the outdoor source, turbulent transport is concentrated within the jet core, associated with intermittent scalar inflow events.

These demonstrate that while both source configurations share common redistribution pathways once the scalar enters the cube, differences in source location lead to distinct patterns of concentration distribution, variance, and the spatial organization of turbulent transport. Such differences emphasize the need to account for source location and the relative roles of advective and turbulent processes when modeling indoor pollutant exposure.

## Summary and conclusion

In urban environments, pollutant ingress from outdoor sources poses a significant challenge to indoor air quality. Cross-ventilation, while essential for passive cooling and natural airflow, can also facilitate the entry of outdoor contaminants into indoor spaces. To gain insight into the outdoor–indoor transport of pollutants in such complex settings, experimental investigations were conducted using an idealized model, namely, a hollow cube representing a scaled-down building, equipped with upstream and downstream window openings, and subjected to an upstream, ground-level passive scalar source. Experiments were carried out in two distinct facilities: a large-scale water tunnel at the University of Southampton, employing Rhodamine dye as a passive scalar (with Schmidt number $Sc \approx 2500\pm 300$), and the EnFlo wind tunnel at the University of Surrey, using propane gas as the scalar ($Sc \approx 1$). In both facilities, the hollow cube was placed within an atmospheric boundary layer. The incoming flow Reynolds number was fixed at $Re = U_{{Ref}}H/\nu \approx 50{,}000$, with a boundary layer thickness-to-building height ratio of approximately 3. The primary objective was to characterize the mean and transient behavior of the scalar field in both outdoor and indoor environments, captured using (simultaneous) planar laser-induced fluorescence (PLIF) and particle image velocimetry (PIV) in the water tunnel, and flame ionization detection (FID) and laser Doppler anemometry (LDA) in the wind tunnel.

After being introduced upstream of the model, the scalar is subsequently transported primarily by streamwise advective flux. Concurrently, a comparatively weaker wall-normal advective flux facilitates its transport in the vertical direction. Throughout this, turbulent fluxes play a role in promoting local mixing of the scalar. As the scalar-laden flow approaches the cube, a portion of the scalar is entrained into the cube through its upstream opening, while the majority circumvents the cube. Within the cube, scalar transport is dominated by a strong advective jet-like flow traversing its midsection. In this central region, turbulent fluxes contribute to scalar homogenization and mixing. Simultaneously, interaction between the jet and the upper and lower recirculation zones (near the roof and ground, respectively) enables scalar exchange across these regions. Some scalar is entrained from the jet into the recirculation zones, while scalar previously trapped in these regions is re-entrained into the jet. Within the recirculation zones themselves, scalar transport is governed primarily by advective fluxes, whereas the exchange between these zones and the central jet region is facilitated by both wall-normal advective and turbulent fluxes. These continuous exchange processes collectively enhance scalar mixing within the cube. Overall, the observations underscore the coupled roles of advective and turbulent mechanisms in governing scalar transport and distribution within and around the cube.

Upon further analysis of the spatial distribution of scalar concentrations indoors, it is observed that the mean (time-averaged) concentration, an indicator of long-term exposure levels, is nearly uniform, indicating a well-mixed environment within the indoor space. This uniformity contrasts with our recent study involving an indoor scalar source (Biswas and Vanderwel 2024), where we reported significant spatial variations in scalar concentrations within the indoor volume, by a factor of up to 20. In addition to analyzing time-averaged concentrations, it is also crucial to investigate the time-varying dynamics of concentration. Upstream of the cube (outdoor), we observe intense fluctuations in concentration, characterized by sharp, intermittent peaks. These peaks represent sporadic scalar parcels carried by the incoming airflow. Similar fluctuations are noted at the inflow window of the cube. Inside the cube, the central area dominated by jets experiences the most significant concentration fluctuations, driven by jet oscillations and the presence of intermittent, highly concentrated scalar parcels. In contrast, the upper and lower recirculation regions show relatively lower concentration fluctuations due to reduced velocity variations and fewer large scalar intrusions from the jet region. Moreover, the fluctuations observed downstream inside the cube and at the outflow window are comparatively lower, indicating that the scalar passing through the cube has undergone a mixing process within it.

In addition to the time trace at different locations, the occurrence of the indoor peak concentrations at these locations is also essential. The ratio of the indoor peak concentration to the concentration fluctuation’s mean-square-root ($C_{99}/c_{rms}$) is seen to be $\approx 5-6$, analogous to the previous studies on indoor dispersions. In the concentration time traces, another important aspect would be the concentration (exponential) decay as $\propto e^{-\tau _dt^{*}}$, which is followed as the source is turned off. The decay constant, $\tau _d$, is seen to scale with wall-normal height, as $\tau _d\propto {{z^{*}}^{-0.16}}$, thus showing a slower concentration decay rate (smaller $\tau _d$) in the upper recirculation region (near the ceiling), and hence indicating a longer exposure risk. These observations are important for understanding peak exposure risks and the corresponding exposure time scales in long-time periods, in the context of ventilation effectiveness.

The current results regarding flow fields, mean and time-varying scalar dynamics, and the time scale of the scalar are largely consistent across both facilities, with only minor differences observed. These differences can be attributed to factors such as the significantly larger dataset available in the wind tunnel. Despite subtle differences, the overall trends and magnitudes of concentration, including its temporal evolution and spatial distribution, align closely across both facilities, despite the substantial difference in Schmidt number (*Sc*) between water and air. This indicates that the turbulent mixing dominates over molecular diffusion in such flows.

Based on the current findings, several possibilities for future research can be followed: $\bullet$ The existing model featuring a centered single window can be further modified to reflect more realistic building designs. $\bullet$ Modifications to the positions of the inlet and outlet windows would significantly alter the indoor airflow patterns, thereby impacting the dynamics of scalar. $\bullet$ Future investigations could also explore scalar injection from elevated sources. $\bullet$ The present results will contribute to future research exploring additional parameters such as buoyancy within more complex geometric configurations.

Overall, this study provides valuable insights into the flow characteristics and mechanisms of passive scalar transport in both indoor and outdoor environments under cross-ventilation, improving our understanding of pollutant exchange, distribution patterns, peak concentrations, and flushing time scales. While real buildings and urban environments involve greater geometric complexity and a wider range of boundary conditions, the present results can inform reduced-order models and parameterizations for larger-scale urban dispersion studies. The identified scalar transport pathways, such as the jet core, recirculation regions, and bulk and turbulent transport, can be incorporated into simplified ventilation and pollutant exchange models, while quantitative metrics, including decay constants, flushing time scales, and peak-to-mean ratios, offer benchmarks for validating and calibrating LES/DNS, RANS-based ventilation simulations, and dispersion models. In addition, the observed exponential relationship governing the infiltration process provides a framework to analyze the lag between outdoor and indoor concentrations and to support source apportionment in real-life scenarios. Collectively, these mechanisms and underlying physics can serve as a baseline for extending insights to more complex configurations, including multi-room buildings, thereby enhancing the applicability of the present findings.

## Data Availability

Data will be made available at the University of Southampton data repository at 10.5258/SOTON/D3672.

## Appendix A

This appendix contains two supplementary tables which provide extended data related to the main results. These have also been referred to in the main text.

**Table 3:** The time-averaged concentration values at: different measurement points (${\overline{C}}^{*}$), line-averaged at the inlet and outlet windows (${\overline{C_l}}^{*}$), and averaged over the entire indoor area (${\overline{C_A}}^{*}$), are shown. Also shown are the standard deviations ($\sigma _{{C}^{*}}$, $\sigma _{{C_l}^{*}}$, and $\sigma _{{C_A}^{*}}$) for the respective instantaneous concentrations (${{C}^{*}}$, ${{C_l}^{*}}$, and ${{C_A}^{*}}$). The standard error associated with these measurements (from bootstrapping) is also shown

Placement	Co-ordinate	Water	Wind	Water	Wind
		$\overline{C}^{*}$	$\overline{C}^{*}$	$\sigma _{{C}^{*}}$	$\sigma _{{C}^{*}}$
	$x^{*}$=-0.25, $z^{*}$=0.25	1.31±0.013	1.11±0.001	0.34±0.01	0.35±0.008
	$x^{*}$=-0.25, $z^{*}$=0.50	1.06±0.041	0.79±0.001	1.27±0.11	0.36±0.009
	$x^{*}$=-0.25, $z^{*}$=0.75	1.11±0.011	0.94±0.0006	0.31±0.02	0.19±0.002
	$x^{*}$=0, $z^{*}$=0.25	1.26±0.014	0.98±0.002	0.39±0.02	0.65±0.017
Indoor	$x^{*}$=0, $z^{*}$=0.50	0.96±0.03	0.62±0.002	0.84±0.07	0.63±0.018
($y^{*}$=0)	$x^{*}$=0, $z^{*}$=0.75	1.12±0.016	0.82±0.0006	0.46±0.09	0.21±0.003
	$x^{*}$=0.25, $z^{*}$=0.25	1.27±0.018	1.09±0.0008	0.52±0.03	0.34±0.008
	$x^{*}$=0.25, $z^{*}$=0.50	1.02±0.021	0.75±0.001	0.64±0.05	0.40±0.010
	$x^{*}$=0.25, $z^{*}$=0.75	1.10±0.012	0.94±0.0004	0.35±0.02	0.23±0.005
	$x^{*}$=0, $z^{*}$=0.25	–	0.91±0.001	–	0.34±0.008
Indoor	$x^{*}$=0, $z^{*}$=0.50	–	0.86±0.001	–	0.41±0.010
($y^{*}$=0.25)	$x^{*}$=0, $z^{*}$=0.75	–	0.86±0.0005	–	0.23±0.005
	$x^{*}$=0, $z^{*}$=0.25	–	0.91±0.001	–	0.35±0.008
Indoor	$x^{*}$=0, $z^{*}$=0.50	–	0.81±0.001	–	0.36±0.009
($y^{*}$=-0.25)	$x^{*}$=0, $z^{*}$=0.75	–	0.81±0.0006	–	0.19±0.002
		$\overline{C_{A}}^{*}$	$\overline{C_A}^{*}$	$\sigma _{{C_A}^{*}}$	$\sigma _{{C_A}^{*}}$
Indoor	Area average	1.09±0.011	–	0.32±0.012	–
		$\overline{C_{l}}^{*}$	$\overline{C_l}^{*}$	$\sigma _{{C_l}^{*}}$	$\sigma _{{C_l}^{*}}$
Inlet ($y^{*}$=0)	$x^{*}$=-0.55, $z^{*}$=0.325-0.675	1.24±0.04	–	1.18±0.05	–
Outlet ($y^{*}$=0)	$x^{*}$=0.55, $z^{*}$=0.325-0.675	1.15±0.019	–	0.52±0.03	–

**Table 4:** The decay constant, $\tau _d$, obtained using Equation 5, is shown for different indoor measurement locations. The decay constants are shown for instantaneous concentrations measured: at different points ($C^{*}$ for $y^{*}=0,0.25,-0.25$); for concentration averaged over indoor area ($C_A^{*}$, in $x-z$ plane at $y^{*}=0$); and line-averaged ($C_l^{*}$ at $y^{*}=0$, at inlet/outlet)

Placement	Co-ordinate	Water tunnel	Wind tunnel
		$\tau _d$	$\tau _d$
	$x^{*}$=-0.25, $z^{*}$=0.25	0.049±0.002	–
	$x^{*}$=-0.25, $z^{*}$=0.50	0.113±0.016	–
	$x^{*}$=-0.25, $z^{*}$=0.75	0.039±0.002	–
	$x^{*}$=0, $z^{*}$=0.25	0.056±0.003	0.078±0.0003
Indoor ($y^{*}$=0)	$x^{*}$=0, $z^{*}$=0.50	0.06±0.004	0.065±0.0003
	$x^{*}$=0, $z^{*}$=0.75	0.042±0.002	0.055±0.0001
	$x^{*}$=0.25, $z^{*}$=0.25	0.051±0.003	–
	$x^{*}$=0.25, $z^{*}$=0.50	0.05±0.005	–
	$x^{*}$=0.25, $z^{*}$=0.75	0.037±0.002	–
	$x^{*}$=0, $z^{*}$=0.25	–	0.070±0.0001
Indoor ($y^{*}$=0.25)	$x^{*}$=0, $z^{*}$=0.50	–	0.058±0.0001
	$x^{*}$=0, $z^{*}$=0.75	–	0.057±0.0001
	$x^{*}$=0, $z^{*}$=0.25	–	0.071±0.0001
Indoor ($y^{*}$=-0.25)	$x^{*}$=0, $z^{*}$=0.50	–	0.063±0.0001
	$x^{*}$=0, $z^{*}$=0.75	–	0.057±0.0001
Indoor ($y^{*}$=0)	Area average	0.037±0.002	–
	Line average at inlet/outlet		
Inlet ($y^{*}$=0)	$x^{*}$=-0.55, $z^{*}$=0.325-0.675	0.048±0.002	–
Outlet ($y^{*}$=0)	$x^{*}$=0.55, $z^{*}$=0.325-0.675	0.102±0.010	–
